# Mechanical, Redox, and Bioelectrical Coupling in Hydrogels for Cutaneous Regeneration: Network Design and Structure–Property Relationships

**DOI:** 10.3390/gels12090818

**Published:** 2026-09-06

**Authors:** Luisbel González, Antonio Pérez-Torres, Yenisleidys Fernández-Guerrero, Daylenis Pérez, Brenda López, Reinier Fernández-López

**Affiliations:** 1 Instituto de Ciencias Aplicadas, Facultad de Ingeniería, Universidad Autónoma de Chile, Santiago 8581151, Chile; brendalp0110@gmail.com; 2 Grupo de Investigación y Desarrollo en Tecnologías Industriales, Universidad Politécnica Salesiana, Cuenca 010105, Ecuador; jperezt@ups.edu.ec; 3 Doctorado en Ingeniería, Universidad del Bío-Bío, Concepción 4051381, Chile; yenisfg94@gmail.com; 4 Laboratorio de Biomateriales, Departamento de Ingeniería Química, Universidad de Concepción, Concepción 4030000, Chile; dayperez@udec.cl; 5 Facultad de Ingeniería y Arquitectura, Universidad Arturo Prat, Iquique 1100000, Chile; rflopez1991@gmail.com

**Keywords:** cutaneous regeneration, wound healing, mechanotransduction, redox signaling, bioelectricity, conductive hydrogels, dynamic networks, cross-domain coupling

## Abstract

Cutaneous wound healing is governed by dynamically interacting mechanical, redox, and bioelectrical signals that regulate cell migration, inflammation, angiogenesis, extracellular-matrix remodeling, and tissue regeneration. Hydrogels are increasingly engineered to modulate these cues; however, most systems are still described through independently measured properties such as stiffness, antioxidant activity, and conductivity, without demonstrating functional coupling among them. This review examines regenerative hydrogels from a cross-domain perspective, integrating the biological basis of mechanotransduction, redox signaling, endogenous bioelectricity, and their molecular convergence with the network-level mechanisms that control hydrogel behavior. Particular emphasis is placed on dynamic crosslinking, viscoelastic relaxation, hydration, redox-active chemistry, ionic and electronic transport, conductive and piezoelectric phases, and degradation-dependent evolution of material function. A conceptual hierarchy is proposed to distinguish property coexistence, structural integration, directional transduction, and adaptive feedback, together with experimental criteria and quantitative approaches for evaluating coupling. Current evidence indicates that mechanoelectrical coupling is the most mature, whereas mechanoredox and redox–electrical interactions remain less systematically quantified. Moving beyond descriptive multifunctionality toward controllable cross-domain transduction may enable hydrogels to function as adaptive soft interfaces capable of responding to the evolving physicochemical conditions of cutaneous regeneration.

## 1. Introduction

Cutaneous wound repair is a highly coordinated regenerative process in which cells continuously interpret biochemical and physical information from a microenvironment that changes throughout healing. Although wound healing is conventionally divided into hemostatic, inflammatory, proliferative, and remodeling phases, this sequential description does not fully capture the simultaneous variations in extracellular-matrix organization, tissue tension, oxidative state, ionic transport, membrane potential, oxygen availability, and intercellular communication that occur after injury. Failure to appropriately resolve these perturbations contributes to persistent inflammation, impaired vascularization, defective matrix remodeling, and incomplete restoration of tissue architecture. Successful regeneration therefore requires not only wound closure but progressive restoration of a microenvironment capable of coordinating migration, immune resolution, angiogenesis, matrix deposition, and re-epithelialization.

Hydrogels are particularly attractive for this purpose because their hydrated polymer networks reproduce several characteristics of the extracellular matrix while providing chemically and mechanically tunable interfaces with damaged tissue. Recent systems have moved well beyond passive moisture retention and drug delivery. Hydrogels combining intrinsic antibiofilm and antioxidant functions have reduced bacterial burden and oxidative stress in infected diabetic wounds [1], while gelatin methacryloyl (GelMA) loaded with platelet lysate promoted angiogenesis and skin regeneration through signal transducer and activator of transcription 3 (STAT3)-associated signaling [2]. These advances demonstrate the versatility of hydrogel platforms but also reflect a prevailing design strategy in which multiple therapeutic functions are often incorporated as parallel modules rather than as interacting physicochemical processes.

This distinction is particularly relevant for mechanical, redox, and bioelectrical regulation. Mechanical resistance and stress relaxation determine how forces are transmitted between the hydrogel, extracellular matrix, and cells; redox reactions regulate both oxidative damage and physiological signaling; and ionic or electronic transport can influence membrane polarization, endogenous electrical gradients, and directional migration. Conductive hydrogel systems have recently demonstrated broader biological effects, including regulation of neutrophil extracellular traps [3] and coordination of peripheral nerve regeneration with the neuro–immune microenvironment in diabetic wounds [4]. Mechanically adaptive conductive hydrogels integrated with self-powered thermoelectric platforms have also been used to distribute microcurrents across wounds and activate regenerative cellular responses [5]. Such findings indicate that conductivity alone is an incomplete descriptor of bioelectrical function because regenerative effects depend on how charge transport interacts with ions, cells, immune signaling, and tissue state.

A similar transition is occurring in redox-active hydrogels. Reactive oxygen and nitrogen species should not be considered exclusively detrimental because their effects depend strongly on concentration, spatial distribution, and healing stage. Accordingly, recent materials increasingly exploit dynamic redox regulation. Reversible silver–catechol chemistry has enabled sustained antioxidant and antibacterial activity [6], whereas ROS-responsive hydrogels containing dimeric copper peptides have combined oxidative-stress regulation with stimulus-dependent therapeutic activity [7]. Closed-loop Pd@Au nanoframe hydrogels further illustrate the transition toward materials capable of linking wound-state sensing with therapeutic response [8]. More recently, quinone-mediated tissue-adaptive double networks have connected catechol oxidation with wet-tissue interactions and mechanical function [9], while sprayable nanozyme hydrogels have remodeled diabetic wound inflammation through multiradical regulation and macrophage reprogramming [10].

Despite this progress, terms such as multifunctional, synergistic, electroactive, and responsive are frequently applied when mechanical properties, antioxidant activity, and conductivity are demonstrated independently. The coexistence of several functionalities, however, does not establish physical or chemical coupling. Functional coupling requires perturbation of one domain to modify the generation, transmission, or biological interpretation of another. Mechanical deformation may reorganize conductive pathways or induce piezoelectric polarization; changes in oxidation state may modify dynamic crosslinks, adhesion, swelling, or electronic transport; ionic redistribution may influence electrical potential and redox reactions; and degradation may simultaneously alter stress transfer, diffusion, and electrical percolation. The relevant design objective is therefore not simply to maximize stiffness, antioxidant capacity, or conductivity, but to control the relationships connecting these properties as the material evolves within the wound.

This review examines regenerative hydrogels from this cross-domain perspective. First, the biological basis of mechanical, redox, and bioelectrical regulation during cutaneous repair is analyzed, with emphasis on the molecular nodes through which these signals converge. The discussion then addresses how hydrogel architecture, crosslinking dynamics, viscoelasticity, hydration, redox chemistry, conductive phases, ionic transport, and degradation generate interdependent material properties. Finally, a framework is proposed to distinguish property coexistence, structural integration, directional transduction, and adaptive feedback. By moving from conventional composition–property–function relationships toward network architecture-cross-domain transduction-evolving physicochemical state-biological response, this review aims to establish a mechanistic basis for designing hydrogels as dynamic soft transduction systems for cutaneous regeneration.

## 2. Mechanical, Redox, and Bioelectrical Regulation of the Cutaneous Wound Microenvironment

Cutaneous wound healing is conventionally divided into hemostatic, inflammatory, proliferative, and remodeling phases. Although this framework remains useful for describing the dominant cellular events after injury, it provides only a partial representation of the information perceived by cells within the wound. Tissue disruption simultaneously modifies extracellular matrix (ECM) continuity, tissue tension, matrix stiffness, oxygen availability, pH, reactive oxygen species (ROS), ionic gradients, membrane potentials, and transepithelial electrical potential. These perturbations occur over different spatial and temporal scales but converge on common processes controlling cell polarity, cytoskeletal remodeling, migration, inflammation, angiogenesis, matrix deposition, and differentiation.

Accordingly, the wound microenvironment should be regarded as a dynamically coupled system rather than as a collection of independent biochemical and physical variables. A distinction between coexistence and coupling is particularly important. Mechanical, redox, and bioelectrical signals coexist when they independently influence cell behavior, whereas functional coupling occurs when perturbation of one domain modifies the magnitude, direction, duration, or spatial organization of another. Several molecular nodes, including integrins, focal adhesion kinase (FAK), Src-family kinases, piezo-type mechanosensitive ion channel component 1 (PIEZO1), Ca^2+^, nicotinamide adenine dinucleotide phosphate (NADPH) oxidases, phosphoinositide 3-kinase (PI3K)/phosphatase and tensin homolog (PTEN), Rho-family GTPases, and the actomyosin cytoskeleton, provide potential routes for such signal integration.

The temporal dimension is equally important. Electrical disruption and ion redistribution occur immediately after injury; Ca^2+^ transients and localized ROS production develop within seconds to minutes; cytoskeletal and focal-adhesion remodeling evolve over minutes to hours; inflammatory and metabolic reprogramming occurs over hours to days; and ECM stiffening, collagen reorganization, tissue contraction, and scar maturation continue for weeks or months. Consequently, a material property that is beneficial during early repair may become maladaptive if it persists during later remodeling. This time dependence provides the biological basis for designing hydrogels that do not simply possess multiple functions but can regulate the wound microenvironment dynamically.

### 2.1. Spatiotemporal Remodeling of the Cutaneous Wound Microenvironment

Immediately after injury, vascular disruption and coagulation generate a fibrin- and fibronectin-rich provisional matrix that differs substantially from native dermal ECM in composition, architecture, and mechanical behavior. This matrix is progressively replaced by granulation tissue containing macrophages, fibroblasts, endothelial cells, newly deposited ECM, and nascent microvessels. Fibroblasts generate traction forces and reorganize collagen, while subsets acquire contractile myofibroblast phenotypes. Importantly, these populations are heterogeneous rather than uniform; distinct fibroblast states contribute differently to ECM deposition, contraction, regeneration, and fibrosis [11].

Changes in ECM composition also modify the ability of cells to perceive mechanical information. For example, wound-associated Agrin enhances keratinocyte mechanoperception, cytoskeletal organization, traction generation, and collective migration through an Agrin–matrix metalloproteinase-12 (MMP12)-dependent mechanism [12]. Such findings illustrate that the ECM acts as an active signaling interface rather than simply as structural support.

At the same time, injury rapidly changes the redox and electrical state of the tissue. Local ROS signals develop shortly after damage, whereas interruption of polarized epithelial ion transport alters transepithelial potential and generates wound-associated ionic currents. Thus, a cell at the wound edge simultaneously experiences changing matrix mechanics, Ca^2+^ fluxes, redox signals, inflammatory mediators, growth factors, and directional electrical cues. Chronic wounds can therefore be interpreted, at least in part, as a failure to restore this multidimensional microenvironment toward physiological conditions. From a biomaterials perspective, the relevant goal is not to maximize an isolated property but to guide the wound through an appropriate sequence of mechanical, redox, and bioelectrical states.

### 2.2. Mechanical Regulation and Mechanotransduction in Cutaneous Repair

Skin is continuously exposed to tensile, compressive, and shear forces generated by tissue prestress, body movement, cellular contraction, and ECM architecture. Injury disrupts this mechanical equilibrium, redistributing tension toward wound margins and replacing native ECM with a provisional matrix that subsequently evolves into collagen-rich granulation tissue. Fibroblast contraction, collagen deposition, fiber alignment, and matrix crosslinking progressively modify local stiffness and stress distribution. The wound therefore represents a dynamic mechanical microenvironment, rather than a substrate characterized by a single constant modulus.

Cells translate mechanical information into biochemical signals through integrins, focal adhesions, cytoskeletal tension, mechanosensitive ion channels, and force-responsive transcriptional regulators. Mechanical loading alone can promote hypertrophic scar formation through altered survival signaling [13], while FAK links mechanical force with extracellular signal-regulated kinase (ERK)/monocyte chemoattractant protein-1 (MCP-1)-associated inflammatory and fibrotic responses [14]. A particularly important downstream mechanism involves yes-associated protein (YAP)-dependent activation of Engrailed-1 (EN1) fibroblasts. Preventing this mechanically induced transition shifted adult murine wound repair away from conventional scarring toward regeneration with restoration of cutaneous appendages [15].

The translational relevance of this pathway has been demonstrated in human fibroblast systems and large-animal wounds. Mechanical strain promotes profibrotic FAK–ERK/YAP states, whereas FAK inhibition preserves protein kinase B (AKT)–early growth response 1 (EGR1)/milk fat globule-EGF 8 (MFGE8)-associated regenerative fibroblast programs and improves collagen organization and tissue biomechanics in red Duroc wounds [16]. Mechanotransduction inhibition also reduces fibrosis and contracture after split-thickness skin grafting [17], and transient targeting of YAP-dependent signaling can promote regenerative rather than fibrotic healing in a large-animal model [18]. These observations distinguish wound closure from true tissue regeneration and identify persistent mechanotransduction as a determinant of scar formation.

Mechanical information is also converted directly into ionic signaling through PIEZO1. In keratinocytes, PIEZO1-dependent Ca^2+^ influx promotes localized retraction and can reduce collective migration efficiency, whereas epidermal *Piezo1* deletion accelerates wound closure [19]. In dermal fibroblasts, however, mechanical stretch increases PIEZO1-mediated Ca^2+^ signaling, proliferation, migration, and myofibroblast differentiation [20]. Matrix stiffening additionally activates PIEZO1-dependent arginine/proline metabolism required for collagen biosynthesis [21] and a Wnt2/Wnt11–C-C motif chemokine ligand 24 (CCL24) pathway that reinforces fibroblast activation and further tissue stiffening [22].

Mechanosensing also influences immune-cell behavior. Macrophages respond to substrate stiffness through PIEZO1-dependent Ca^2+^ signaling [23], and graded mechanical stretch produces non-monotonic macrophage responses, with intermediate loading producing more favorable reparative effects than either weaker or stronger stimulation [24].

**Figure 1:**
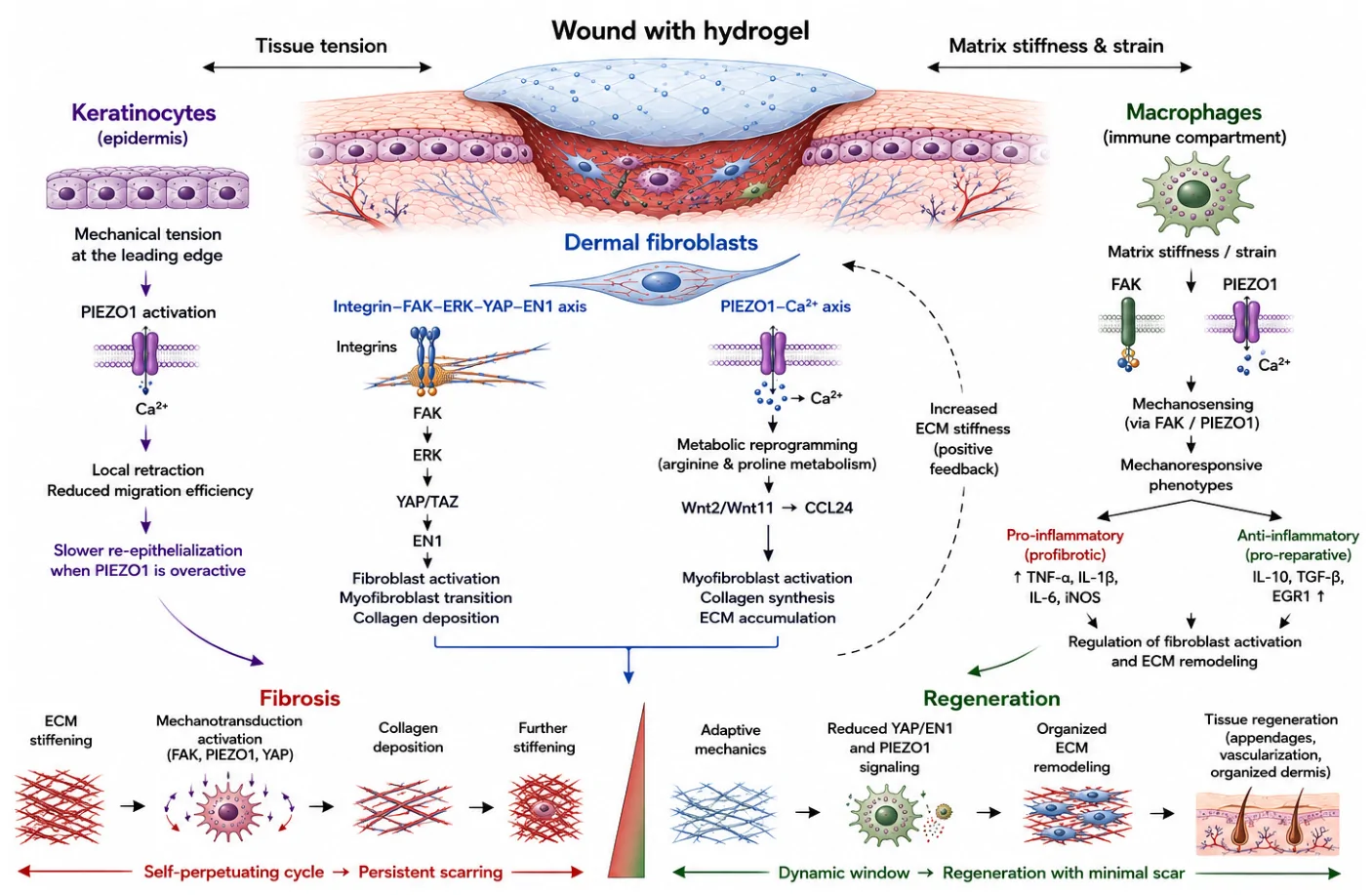
Evidence-informed model of the mechanical microenvironment and cell-specific mechanotransduction during cutaneous wound healing. Solid arrows represent experimentally supported mechanistic relationships, whereas dashed arrows indicate conceptual integration of multiple pathways into the overall balance between fibrosis and regeneration. The diagram does not imply that all molecular nodes constitute a single linear signaling pathway. Mechanistic relationships are based on the evidence discussed in Section 2.2 and recent comprehensive analyses of cutaneous wound healing and fibroblast/myofibroblast mechanobiology [25,26].

Mechanical signals are therefore highly dependent on cell identity, magnitude, duration, and tissue context (Figure 1 and Table 1). Figure 1 integrates two levels of evidence. Solid connections represent experimentally supported mechanotransduction relationships discussed above, including FAK-dependent force sensing, PIEZO1-mediated Ca^2+^ signaling, and YAP/TAZ-associated fibroblast activation. In contrast, dashed connections toward the global fibrosis–regeneration outcome represent a conceptual synthesis of how these cell-specific mechanisms may collectively influence tissue repair rather than a single experimentally demonstrated signaling cascade [25,26].

For hydrogel design, these observations argue against maximizing stiffness, tensile strength, toughness, or adhesion as independent objectives. The material should initially provide sufficient integrity and support for cell traction while subsequently permitting stress dissipation, cellular infiltration, ECM remodeling, and resolution of profibrotic signaling. Self-adaptive hydrogels capable of modulating YAP–TEA domain transcription factor (TEAD)/EN1-associated mechanotransduction according to healing stage illustrate this transition from static mechanical matching toward temporally programmed mechanoregulation [27]. The emerging objective is therefore a mechanically permissive and adaptive wound interface that supports repair without sustaining FAK–YAP–PIEZO1-associated fibrotic feedback.

**Table 1:** Representative primary studies defining mechanical signaling and mechanotransduction mechanisms relevant to cutaneous wound healing and skin regeneration.

Model	Signal/Intervention	Molecular Mechanism	Biological Evidence	Implications for Hydrogel Design	Evidence Level
Murine cutaneous wounds [13]	Increased mechanical loading	Mechanical load → Akt signaling → decreased apoptosis	Mechanical loading during the proliferative phase was sufficient to induce hypertrophic scar formation, increasing scar cellularity and tissue accumulation.	Early causal evidence that mechanical loading alone can redirect wound repair toward pathological fibrosis.	A
Mouse wounds; fibroblasts [14]	Mechanical loading; FAK inhibition/deletion	FAK → ERK → MCP-1 inflammatory signaling → fibrosis	FAK was activated after cutaneous injury and potentiated by mechanical loading; disruption of FAK signaling reduced inflammation and fibrosis.	Establishes FAK as a mechanistic interface between mechanical force, inflammation, and scar formation.	A/C/G
Mouse wounds; fibroblast lineage tracing [15]	Mechanical tension; YAP inhibition	Mechanical tension → YAP → EN1 fibroblast activation	Blocking YAP-dependent EN1 activation shifted adult wound healing from conventional scarring toward regenerative repair with restoration of skin appendages.	Strong causal evidence that mechanotransduction regulates the fibrosis-versus-regeneration decision, rather than wound closure alone.	A/G
Human fibroblasts in 3D collagen matrices; red Duroc pig wounds [16]	Mechanical strain; pharmacological FAK inhibition	FAK inhibition → ↓mitogen-activated protein kinase (MAPK)–ERK/YAP; preserved AKT → EGR1/MFGE8	Mechanical strain generated heterogeneous profibrotic fibroblast states, whereas FAK inhibition suppressed fibrotic programs and promoted regenerative fibroblast trajectories, hair-follicle recovery, normal collagen architecture, and improved biomechanics.	Strong translational evidence connecting mechanical signaling with opposing fibrotic and regenerative fibroblast states.	H/C/LA/BM
Keratinocytes; epidermal *Piezo1* mouse model [19]	PIEZO1 activation/inhibition	PIEZO1 → Ca^2+^ → local cellular retraction	PIEZO1 promoted local keratinocyte retraction and reduced migration efficiency; epidermal *Piezo1* deletion accelerated wound closure.	Demonstrates that mechanosensing is strongly cell- and context-dependent and that increased mechanosensor activity is not necessarily pro-regenerative.	A/C/G
Human dermal fibroblasts; rat hypertrophic scars [20]	Cyclic mechanical stretch	Stretch → PIEZO1 → Ca^2+^ influx → fibroblast activation	Mechanical stretch increased PIEZO1 expression and Ca^2+^ influx and promoted fibroblast proliferation, migration, and myofibroblast differentiation; PIEZO1 inhibition attenuated hypertrophic scarring.	Direct mechanical-to-ionic coupling through a mechanosensitive channel, providing an important link with electrochemical signaling.	H/C/A/G
Macrophages cultured on matrices of different stiffness; wound-related models [23]	Matrix stiffness; PIEZO1 modulation	Stiffness → PIEZO1 → Ca^2+^ signaling → macrophage polarization	PIEZO1 acted as a sensor of substrate stiffness and regulated macrophage inflammatory polarization; loss of PIEZO1 reduced inflammatory responses and favored wound-healing phenotypes.	Demonstrates that matrix mechanics can regulate the immune microenvironment through mechanically activated ion signaling.	C/G
Red Duroc split-thickness skin graft model [17]	Local inhibition of mechanotransduction	FAK-dependent mechanotransduction	Mechanotransduction blockade decreased fibrosis and contracture in split-thickness skin grafts and improved tissue remodeling.	Large-animal evidence that modifying force sensing influences long-term structural repair rather than merely wound closure.	LA/BM
Dermal fibroblasts on soft versus stiff matrices; mouse skin fibrosis [21]	Matrix stiffness; PIEZO1 targeting	Stiffness → PIEZO1 → arginase 1 (Arg1)/ornithine aminotransferase (OAT)/pyrroline-5-carboxylate reductase 1 (PYCR1)→ arginine/proline metabolism	Increased matrix stiffness enhanced arginine and proline metabolism, providing metabolic substrates for collagen biosynthesis; PIEZO1 inhibition disrupted this response and reduced fibrosis.	Provides a direct mechanistic connection between matrix mechanics, ion-channel activation, metabolism, and ECM production.	C/A/G
Human and mouse fibrotic skin; dermal fibroblasts [22]	Increased matrix stiffness	PIEZO1 → Wnt2/Wnt11 → CCL24 → fibroblast activation	Stiffness activated a PIEZO1-dependent Wnt2/Wnt11–CCL24 profibrotic pathway; adeno-associated virus (AAV)-mediated *Piezo1* knockdown reduced myofibroblast activation, fibrosis, and skin stiffness.	Establishes a self-reinforcing stiffness → mechanotransduction → fibrosis → further stiffness circuit.	H/A/C/G
THP-1-derived macrophages; keratinocyte, fibroblast and endothelial-cell assays; full-thickness wound model [24]	Static stretch at 7%, 15%, and 21%	Mechanical stretch → YAP/transcriptional coactivator with PDZ-binding motif (TAZ)-dependent macrophage response	Macrophage responses were non-monotonic; 15% stretch produced the strongest reparative response, enhanced M2-associated signaling, and conditioned media that promoted migration, angiogenesis, and dermal reconstruction.	Strong evidence that mechanical effects follow a biological response window, rather than a linear “more force–better healing” relationship.	C/A
Self-adaptive mechanoregulative hydrogel; skin wound model [27]	Stage-dependent control of wound mechanics	Mechanical niche regulation → YAP–TEAD → EN1^+^ fibroblast modulation	A dynamically adaptive hydrogel modulated the mechanical niche according to wound-healing stage and promoted rapid repair with reduced scar formation.	Represents the transition from fixed mechanical properties toward temporally programmed mechanoregulation in hydrogel design.	A/BM
Red Duroc large-animal wounds [18]	Transient YAP mechanotransduction inhibition	Mechanical signaling → YAP-dependent fibroblast fate	A single transient intervention targeting YAP-dependent mechanotransduction prevented conventional scar formation and promoted regenerative wound healing in a large-animal model.	Strong translational confirmation that mechanical signaling regulates the balance between scar formation and tissue regeneration.	LA/G

Evidence level: H, human tissue/clinical physiology; LA, large-animal model; A, animal model; C, cellular/in vitro; G, genetic or molecular causal perturbation; BM, biomaterial-mediated intervention; NM, non-mammalian mechanistic model.

### 2.3. Redox Signaling and Redox Homeostasis in Cutaneous Repair

ROS are frequently treated in wound biomaterials as deleterious species that should be eliminated; however, this interpretation does not reflect their physiological signaling functions. Superoxide (O_2_•^−^), H_2_O_2_, hydroxyl radicals (•OH), and related reactive species differ substantially in lifetime, diffusion range, reactivity, and molecular targets. H_2_O_2_ is particularly important because it can act as a relatively stable second messenger through reversible oxidation of redox-sensitive cysteine residues. Experimental wound models demonstrate a biphasic response: low or controlled H_2_O_2_ concentrations can support angiogenesis and repair, whereas excessive exposure delays healing; conversely, excessive catalase-mediated removal of endogenous H_2_O_2_ can also suppress angiogenesis and wound closure [28]. Similarly, 10 mM topical H_2_O_2_ enhanced angiogenesis and closure in murine excisional wounds, whereas 166 mM delayed repair and prolonged inflammatory remodeling [29]. These findings support a redox signaling window rather than a simple relationship between lower ROS and better healing.

The spatial organization of ROS is equally important. Niethammer et al. demonstrated that epithelial injury generates a dual oxidase (DUOX)-dependent H_2_O_2_ gradient within minutes of wounding, providing positional information for leukocyte recruitment [30]. Yoo et al. subsequently identified oxidation of Lyn kinase at Cys466 as one mechanism capable of converting wound-derived H_2_O_2_ into leukocyte chemotactic signaling [31]. Although these studies were performed primarily in zebrafish, they established the fundamental concept that ROS can encode spatial information and act through specific redox-sensitive molecular targets rather than functioning only as nonspecific oxidants.

Redox signaling is also linked to ionic and mechanical events. Injury-induced Ca^2+^ flashes can activate DUOX and initiate H_2_O_2_ production [32], thereby connecting Ca^2+^ signaling with the early oxidative response. More recent high-resolution experiments identified localized dual oxidase 2 (DUOX2)-derived H_2_O_2_ flashes associated with actin remodeling and epithelial migration. Importantly, a PIEZO1–DUOX2–FER tyrosine kinase (FER)/cortactin pathway connected mechanosensing, Ca^2+^-related signaling, localized oxidation, and cytoskeletal dynamics [33]. This provides direct evidence that redox signaling can participate in a mechanoredox circuit rather than operating as an isolated biochemical pathway.

Persistent oxidative stress represents a fundamentally different state. Chronic hyperglycemia impairs Kelch-like ECH-associated protein 1 (KEAP1)–nuclear factor erythroid 2-related factor 2 (NRF2) signaling and weakens endogenous antioxidant responses, contributing to ROS accumulation and defective diabetic tissue regeneration. Restoration of NRF2 activity through KEAP1 suppression re-establishes redox homeostasis and improves regenerative outcomes [34]. NRF2 also influences intercellular communication: epidermal NRF2 regulates a C-C motif chemokine ligand 2 (CCL2)-dependent macrophage recruitment pathway that promotes macrophage-derived epidermal growth factor (EGF) and keratinocyte proliferation [35]. Thus, redox regulation influences not only oxidative damage but also multicellular coordination within the wound.

Importantly, increased expression of a ROS-generating enzyme does not necessarily establish causal dependence. Genetic deletion of NADPH oxidase 4 (NOX4) did not substantially impair physiological cutaneous wound healing or transforming growth factor beta (TGF-β)-induced fibroblast-to-myofibroblast differentiation despite marked NOX4 induction during profibrotic stimulation [36]. This observation highlights a methodological limitation relevant to antioxidant hydrogels: reductions in fluorescent ROS probes or high values obtained using 2,2-diphenyl-1-picrylhydrazyl (DPPH), 2,2′-azino-bis(3-ethylbenzothiazoline-6-sulfonic acid) (ABTS), or ferric reducing antioxidant power (FRAP) assays do not by themselves demonstrate restoration of physiologically meaningful redox signaling.

The evidence summarized in Table 2 therefore supports a design objective based on dynamic redox buffering rather than maximal ROS scavenging. Hydrogel networks should limit sustained oxidative stress while preserving localized ROS-dependent processes required for damage sensing, migration, angiogenesis, and immune coordination. This may ultimately be achieved through controlled diffusion, reversible redox-active chemistry, catalytic regulation, or stimulus-responsive network transformations. The redox permissive window thus constitutes the second component of the integrated framework developed in this review.

### 2.4. Endogenous Bioelectrical Signaling and Electrotaxis in Cutaneous Repair

Intact skin maintains a transepithelial electrical potential through polarized ion transport and selective membrane permeability. Following injury, local disruption of the epidermal barrier creates a low-resistance pathway while surrounding intact tissue continues to maintain ion transport, generating an injury current and lateral electric field directed toward the wound. These endogenous fields are physiologically relevant in humans. Non-invasive measurements adjacent to human skin wounds detected substantial lateral electric fields and showed that their magnitude decreased with age [40].

Keratinocytes are highly sensitive to electric fields within the range measured near wounds. Primary human keratinocytes migrate essentially randomly at fields ≤5 mV/mm but show increasingly directional cathodal migration between approximately 10 and 100 mV/mm [41]. This electrotactic response depends on extracellular Ca^2+^ and growth-factor signaling [42] and involves pathways shared with mechanotransduction, including β4-integrins, EGF signaling, Rac family small GTPase 1 (Rac1), focal adhesions, and cytoskeletal polarization [43].

The PI3K/PTEN axis provides particularly strong causal evidence that wound electrical fields act as instructive signals. Physiological electric fields polarize intracellular signaling, while genetic disruption of PI3Kγ impairs electrotaxis and PTEN deletion enhances electrically guided responses; experimentally altering endogenous electrical signals also changes wound repair in vivo [44]. Electrical cues can additionally regulate vascular responses. Physiologically scaled fields stimulate endothelial-cell orientation, directional migration, and vascular endothelial growth factor (VEGF)-associated signaling through PI3K/AKT and Rho/Rho-associated coiled-coil-containing protein kinase (ROCK) pathways [45].

As with mechanical and redox signals, however, electrical stimulation is strongly context-dependent. Human dermal fibroblasts exposed to electrical stimulation can display increased proliferation and migration but also enhanced matrix contraction and myofibroblast differentiation [46]. Moreover, keratinocytes and fibroblasts can respond differently to the same electrical field in co-culture [47]. Therefore, a single electrical intensity cannot be assumed to be universally regenerative across the multicellular wound environment.

This evidence also exposes a critical distinction for conductive hydrogel research: electrical conductivity is a material property, whereas bioelectrical signaling is a biological function. Reporting conductivity in S/m does not demonstrate that a hydrogel preserves endogenous wound currents, transmits a physiologically relevant electric field, induces electrotaxis, modulates ion-channel activity, or activates regenerative polarity pathways. Consequently, conductive and electroactive hydrogels should be evaluated using functional outcomes such as hydrated impedance, field magnitude, current density, stability under deformation, cell polarization, Ca^2+^ redistribution, electrotaxis, and downstream tissue responses. The principal evidence supporting this bioelectrical signaling window is summarized in Table 3.

### 2.5. Molecular and Physical Coupling of Mechanical, Redox, and Bioelectrical Signals

The evidence described above indicates that mechanical, redox, and bioelectrical cues share multiple molecular and physical interfaces. Their simultaneous presence, however, should not automatically be interpreted as coupling. For the purposes of this review, functional coupling is defined as a condition in which a change in one domain modifies the generation, transmission, or biological interpretation of another. This definition distinguishes genuinely coupled systems from conventional multifunctional hydrogels in which mechanical strength, antioxidant activity, and conductivity are independently measured but no causal relationship between them is demonstrated.

Mechanoredox coupling can occur when mechanical deformation modifies intracellular Ca^2+^ signaling and ROS generation or, conversely, when localized redox chemistry alters cytoskeletal force production. The PIEZO1–DUOX2–H_2_O_2_–FER/cortactin pathway described during epithelial repair provides direct molecular evidence for such integration [33]. More generally, Ca^2+^ provides a plausible convergence node because it is generated downstream of mechanically gated channels while simultaneously regulating ROS-producing enzymes and cytoskeletal dynamics.

Mechanobioelectric coupling is physically even more direct because membrane deformation can regulate ion-channel activity and therefore alter charge distribution and membrane potential. In vivo evidence outside the cutaneous system has demonstrated that tissue stretching can generate endogenous electric fields through stretch-sensitive channels and that these fields can direct collective migration [54]. Although this developmental *Xenopus* model should not be interpreted as direct skin-wound evidence, it establishes an important physical principle for regenerative materials. In wound repair itself, a recent biomimetic system combining a mechanically regulated environment with a directional physiological electric field enhanced keratinocyte migration, re-epithelialization, and organized ECM regeneration through PIEZO1-associated mechanisms [55].

Piezoelectric hydrogels provide a material-level implementation of the same principle by converting deformation into local electrical output. For example, a piezoelectric, conductive, injectable hydrogel capable of harvesting biomechanical deformation generated local electrical stimulation and improved wound repair [56]. Such systems differ fundamentally from passive conductive hydrogels because mechanical changes actively modify their electrical state.

Redox–bioelectric coupling remains less extensively demonstrated but is particularly relevant to redox-active conductive materials. Ag/Zn redox-active bioelectric dressing simultaneously generated electrical activity and H_2_O_2_-dependent signaling that enhanced human keratinocyte migration; attenuation of ROS reduced this response [57]. The mechanistic principle is relevant beyond metallic dressings. Oxidation state, electron transfer, and charge transport are intrinsically linked in conducting polymers, graphene-derived systems, and catechol/quinone-containing networks. Nevertheless, in most hydrogel studies, antioxidant activity and conductivity continue to be measured independently, and direct evidence that redox transformations regulate electrical behavior—or vice versa—remains limited.

Taken together, these studies suggest three progressively stronger levels of evidence. At the lowest level, coexistence describes materials that independently exhibit mechanical, redox-active, and conductive properties. Pairwise coupling requires demonstration that one domain directly changes another, such as deformation-dependent electrical output or redox-dependent cytoskeletal remodeling. The strongest level, integrated coupling, requires a causal chain linking mechanical, redox, and bioelectrical processes to cellular signaling and tissue regeneration. Evidence at this third level remains scarce, representing one of the major research gaps identified in this review. Representative experimental evidence supporting these different modes of cross-domain coupling is summarized in Table 4.

The collective evidence therefore supports a mechanoredox–bioelectric permissive window rather than independent optimization of stiffness, antioxidant capacity, and conductivity. Within this framework, mechanical properties should support adhesion and migration without maintaining profibrotic mechanotransduction; redox-active components should buffer pathological oxidative stress without suppressing physiological ROS signaling; and electrical properties should facilitate biologically relevant charge and ionic communication rather than merely maximize conductivity. This conceptual transition—from multifunctionality to controlled functional coupling—provides the basis for the following section, which focuses on how hydrogel network chemistry, architecture, and composition can be engineered to generate these coordinated responses.

**Figure 2:**
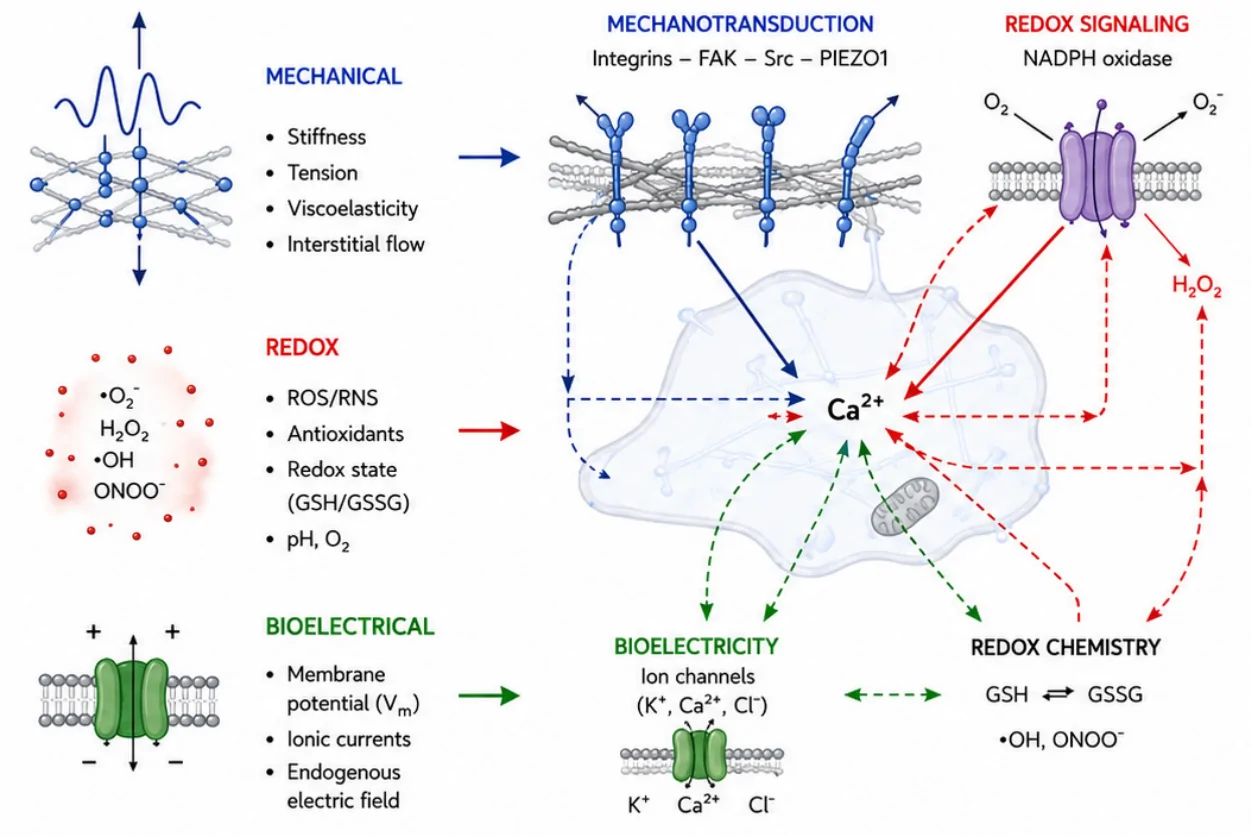
Evidence-informed integration of mechanochemical, redox, and bioelectrical signaling in the cutaneous wound microenvironment. Solid arrows indicate molecular relationships supported by direct mechanistic evidence discussed in Section 2, whereas dashed arrows denote conceptual or indirect cross-domain connections synthesized in this review. The diagram is intended to identify potential points of convergence rather than to represent a single universal signaling pathway [25,26].

The principal molecular interfaces linking mechanical, redox, and bioelectrical signaling across the wound microenvironment are summarized in Figure 2. The figure should be interpreted as an evidence-informed integrative map rather than as a single experimentally validated signaling cascade. Solid connections identify molecular relationships for which direct mechanistic evidence is discussed in Section 2, whereas dashed connections represent broader cross-domain convergence inferred from the combined literature and used here to organize the proposed coupling framework [25,26].

## 3. Hydrogel Network Architecture and Interdependence of Mechanical, Redox, and Electrical Properties

The mechanoredox–bioelectrical processes described in Section 2 originate, at the material level, from the molecular architecture and physicochemical state of the hydrogel. In a hydrated polymer network, mechanical resistance, viscoelastic relaxation, molecular diffusion, redox activity, ionic mobility, electronic transport, degradation, and interfacial adhesion cannot generally be varied independently. They arise from a common set of structural variables, including polymer volume fraction, effective crosslink density, network topology, crosslink lifetime, chain mobility, mesh size, water content, distribution of functional groups, and the organization of conductive or catalytic phases. Consequently, modification of one network parameter can alter several properties simultaneously. Increasing effective crosslink density may improve dimensional stability but restrict chain rearrangement and solute diffusion; increasing hydration generally facilitates ionic transport while decreasing the polymer fraction available to support mechanical load; oxidation of catechol-containing groups may alter both radical-scavenging capacity and intermolecular interactions; and swelling or deformation of a nanocomposite network may modify the distance between conductive domains and therefore its electrical resistance.

This interdependence has important consequences for the interpretation of multifunctional wound hydrogels. The simultaneous measurement of an elastic modulus, antioxidant activity, and electrical conductivity demonstrates the coexistence of three properties, but not necessarily their physical or chemical coupling. Evidence of coupling requires showing that perturbation of one material domain changes another—for example, that deformation changes electrical output, oxidation alters network mechanics or conductivity, or mechanical activation controls ROS production. This distinction becomes increasingly important as hydrogel design shifts from the incorporation of multiple independent functionalities toward dynamically interacting networks capable of changing their state during wound repair.

### 3.1. Network Architecture, Crosslinking, and Dynamic Rearrangement

Hydrogel properties are initially established by the connectivity and topology of the polymer network. In an idealized permanently crosslinked network, elastic resistance is primarily related to the density of elastically active chains; however, biological hydrogels deviate substantially from ideal network behavior because loops, dangling chains, heterogeneous junctions, physical entanglements, solvent redistribution, reversible associations, and network defects also contribute to the measured response. Therefore, nominal polymer concentration or crosslinker content cannot be assumed to be equivalent to an effective crosslink density. This consideration is particularly relevant when comparing wound hydrogels from different studies, in which apparently similar storage moduli may arise from very different molecular mechanisms.

Permanent covalent networks provide long-lived structural connectivity and are advantageous when prolonged dimensional stability, resistance to dissolution, or controlled shape retention is required. Photopolymerized methacrylate networks, free-radical polymerized acrylamide derivatives, amide-coupled networks, and other irreversible covalent systems therefore remain widely used. Their principal limitation is that stress imposed by cells or tissue movement cannot readily be dissipated through junction exchange. Relaxation instead depends on solvent redistribution, polymer-chain rearrangement, irreversible network damage, or degradation. As a result, increasing covalent crosslink density can simultaneously improve structural retention and increase the persistence of mechanical stress transmitted to cells.

Dynamic covalent networks introduce an additional time scale because individual junctions can dissociate and reform. Imine bonds, hydrazones, boronate esters, disulfides, and related reversible interactions have consequently been incorporated into injectable and self-healing wound hydrogels. Importantly, dynamic bonding, self-healing, injectability, and stress relaxation should not be treated as synonymous properties. Macroscopic self-healing evaluates recovery after deliberate network disruption, whereas stress relaxation describes the decay of stress during sustained deformation. Both depend partly on bond exchange, but they interrogate different physical processes.

Dynamic interpenetrating networks are particularly valuable because distinct subnetworks can provide complementary functions. An extracellular-matrix-mimetic interpenetrating hydrogel in which a more persistent GelMA-based network was combined with a dynamically reorganizing hyaluronic-acid-derived component, allowing structural stability and viscoelastic adaptation to be partially separated [64]. More recently, double-crosslinked HA–gelatin–oxidized dextran systems combining irreversible amide junctions with reversible imine bonds demonstrated that adjustment of dynamic network chemistry can modify modulus, relaxation behavior, yield properties, and regenerative performance without requiring complete loss of network integrity [65].

This architectural distinction becomes even more relevant when conductive or redox-active phases are introduced. A functional nanoparticle or polymer does not simply add a new property to an otherwise unchanged matrix. Its surface chemistry can create additional physical or coordination junctions, modify local polymer density, alter pore architecture, influence water uptake, and provide new pathways for load transfer. Improved dispersion of heparin–polydopamine-reduced graphene oxide within a polyacrylamide network increased compressive and tensile properties while simultaneously producing a continuous electrically conductive phase. The optimized hydrogel reached tensile strengths of approximately 250 kPa and conductivities up to 3.63 S/m, illustrating how nanoscale dispersion affects mechanical and electrical behavior concurrently [66].

Thus, network architecture should be described not only by polymer identity and crosslinking reaction but by the resulting hierarchy of load-bearing, dissipative, redox-active, and charge-transporting elements. For the present framework, the most relevant architectures are those in which these elements occupy overlapping structural domains, because such organization creates the physical basis for coupling among mechanical, redox, and electrical behavior.

### 3.2. Time-Dependent Mechanics and Mechanical Adaptation

Mechanical characterization of wound hydrogels is frequently reduced to Young’s modulus, compressive modulus, tensile strength, or storage modulus (G′). Although these measurements are necessary, they provide an incomplete description of the mechanical signal transmitted to the wound. Skin, provisional ECM, and granulation tissue are viscoelastic systems in which stress changes continuously through fluid redistribution, cellular contraction, collagen remodeling, proteolysis, and matrix deposition. Accordingly, a hydrogel that initially matches the modulus of skin may subsequently impose an inappropriate mechanical environment if it relaxes too slowly, stiffens during degradation or oxidation, or remains mechanically dominant after the regenerating tissue begins to carry load.

The distinction between stiffness and stress relaxation is particularly important. Two hydrogels with similar instantaneous moduli can transmit markedly different forces to cells when the lifetime of their junctions differs. In dynamic networks, faster exchange of reversible bonds permits local polymer rearrangement around cellular traction forces and decreases sustained resistance to deformation. Conversely, slowly exchanging or permanent junctions preserve stress for longer periods. These differences are directly relevant to the FAK–YAP–PIEZO1 pathways discussed in Section 2, because cellular mechanotransduction depends on the ability of the surrounding material to resist and redistribute traction rather than on modulus alone.

The HA–gelatin–dextran system provides a useful recent example. By combining stable amide crosslinks with dynamic imine interactions, network relaxation could be programmed while retaining sufficient structural integrity, demonstrating that low stiffness and high deformability need not necessarily imply mechanical instability [65]. Such results reinforce the need to characterize regenerative hydrogels through time-dependent metrics including G′, G″, loss tangent (tan δ), stress-relaxation kinetics, creep and recovery, cyclic hysteresis, fatigue, fracture resistance, and changes in these parameters under hydrated physiological conditions.

Nanocomposite systems add another source of mechanical complexity because functional fillers can simultaneously reinforce and reorganize the network. In the Hep–PDA–rGO/PAM system, homogeneous distribution of the graphenic phase improved load transfer and increased tensile and compressive resistance; excessive PDA incorporation, however, decreased mechanical strength, indicating that increasing the concentration of a functional component does not necessarily produce monotonic mechanical improvement [66]. This finding is conceptually important for multifunctional design: concentrations optimized for redox activity or conductivity may not coincide with concentrations that optimize mechanical performance.

A more explicit integration of mechanics with other functions is evident in recent thermoresponsive and piezoelectric networks. A double-network dressing containing a physically crosslinked ureidopyrimidinone-modified gelatin network and a chemically polymerized N-isopropylacrylamide/GelMA network. The material combined high mechanical strength with temperature-dependent softening and active contraction, while incorporated BTO@Au generated piezoelectric electrical output and ROS in response to ultrasound [58]. Here, mechanical functionality is no longer simply passive structural support: network contraction becomes an active component of treatment, while mechanical deformation of the embedded piezoelectric phase controls other physicochemical outputs.

This represents an important shift in design philosophy. For wound regeneration, an advanced hydrogel should not necessarily maintain constant mechanical properties throughout treatment. More physiologically relevant systems may require initial cohesion and adhesion, followed by progressive relaxation, remodeling, or weakening as newly deposited tissue assumes mechanical function. This requirement makes mechanical evolution, rather than initial stiffness alone, a central structure–property parameter.

### 3.3. Redox-Active and Redox-Responsive Network Chemistry

The redox properties of hydrogels arise through several fundamentally different mechanisms and should therefore be classified according to their chemical basis. A hydrogel may contain redox-active groups capable of reversible electron or hydrogen transfer, ROS-scavenging molecules that are progressively consumed, catalytic nanozymes that transform reactive species, or ROS-responsive bonds that undergo chemical cleavage. These mechanisms produce different kinetics and different consequences for network structure.

Polyphenols are particularly attractive because their redox chemistry is intrinsically coupled to intermolecular interactions. Catechol and gallol groups can participate in hydrogen bonding, aromatic interactions, coordination with metals, radical reactions, and oxidation to quinone species. Their redox state therefore influences more than antioxidant capacity. Oxidation can change hydrogen-bonding ability, coordination geometry, electrophilicity, interfacial adhesion, and the formation of covalent bonds with nucleophiles. In a hydrogel in which catechol-containing groups participate directly or indirectly in crosslinking, redox transformation can consequently modify network architecture.

This principle is relevant to the CMCS/THB/Cu/GB hydrogels, which combined dynamic interactions responsible for self-healing and mechanical stability with polyphenol-derived antioxidant behavior and graphene-based conductivity. The same formulation was also capable of photothermally controlled nitric oxide release, demonstrating that chemical composition simultaneously determined redox, mechanical, and electrical functions [67]. Importantly, however, simultaneous presence of these properties does not prove that the redox state directly changes electrical conduction or mechanical behavior. This distinction should be preserved when such systems are described as multifunctional.

The PDA–Fe–PEDOT nanozyme hydrogel developed by Ran et al. [60]. provides a more informative example of interaction between redox and electrical domains. Dopamine-grafted fish gelatin and methacrylated silk fibroin formed the hydrogel matrix, while PDA–Fe–PEDOT nanozymes supplied electrical conductivity together with peroxidase-like activity and sustained antioxidant behavior. Notably, antioxidant capacity remained detectable during exogenous electrical stimulation, while the conductive nanozyme was capable of both H_2_O_2_-dependent catalytic ROS generation and long-term radical scavenging [60]. This system is particularly relevant because redox and electronic functions arise from the same hybrid nanozyme rather than from spatially unrelated additives.

Tannic acid/poly(3,4-ethylenedioxythiophene):poly(styrene sulfonate) (PEDOT:PSS) systems provide another chemically interesting architecture. A recent sodium alginate/tannic acid/PEDOT:PSS hydrogel combined calcium-mediated network formation, phenolic redox activity, and mixed conductive behavior within the same hydrated matrix [68]. Such systems are potentially valuable for studying redox–electrical coupling because PEDOT conductivity depends on electronic structure and doping state while polyphenols can participate in electron-transfer chemistry and polymer interactions. Nevertheless, demonstrating this coupling requires direct measurement of conductivity or impedance as a function of controlled oxidation/reduction state; antioxidant activity and conductivity measured independently remain evidence of coexistence rather than causality.

An additional consideration is that ROS generation can be desirable rather than pathological when it is spatially and temporally controlled. Piezoelectric catalytic hydrogels illustrate this particularly clearly. In the BTO@Au system of Luo et al. [58], ultrasound-induced mechanical deformation generated electron–hole separation and surface polarization. At high ultrasound power, the resulting redox reactions produced bactericidal ROS; at lower power, lower ROS output was accompanied by sustained local electrical stimulation that supported cellular migration and angiogenesis [58]. Redox regulation in this context therefore means controlling ROS flux, not simply maximizing scavenging capacity.

A similar principle appears in the LIFU-responsive BT-dNO@Gel. Mechanical excitation of the barium titanate-based component generated surface charge, local direct current, and piezocatalytic ROS, while the same external stimulus triggered nitric oxide release. Electron-spin-resonance measurements confirmed formation of •OH and O_2_•^−^, whereas the hydrogel’s mechanical properties provided adhesion, self-healing, swelling capacity, and wound coverage [63]. This study is among the strongest current examples of a system in which redox output is physically linked to mechanical energy conversion rather than merely being incorporated as an independent antioxidant component.

These findings also highlight a methodological limitation in the field. DPPH, ABTS, or single-time-point intracellular ROS measurements can establish chemical or cellular antioxidant activity but provide limited information about the kinetics, reversibility, spatial distribution, and functional lifetime of redox behavior. A rigorous characterization of redox-active hydrogels should increasingly examine defined ROS, time-dependent H_2_O_2_ consumption or generation, repeated oxidative challenges, chemical-state changes, catalytic cycling, and the effect of oxidation/reduction on swelling, mechanics, adhesion, and electrical properties.

### 3.4. Electrical Transport and Electromechanical Conversion

Electrical functionality in hydrated hydrogels must be interpreted according to the mechanism of charge transport. Ionic conductivity results primarily from migration of solvated ions through the aqueous phase and therefore depends on ion concentration, water fraction, polymer–ion interactions, tortuosity, and effective mesh dimensions. Electronic conductivity requires sufficiently connected electronically active domains such as PEDOT, polypyrrole, graphene derivatives, carbon nanotubes, MXenes, metallic nanostructures, or semiconductor phases. Many wound hydrogels exhibit mixed ionic/electronic conduction, but these contributions are rarely separated experimentally.

A more standardized characterization workflow is therefore required for mixed ionic–electronic conductive hydrogels. As a minimum, total transport should first be characterized by electrochemical impedance spectroscopy (EIS) under a defined hydrated state, using a reported electrode material, electrode area and spacing, electrolyte composition, temperature, AC perturbation amplitude, and frequency range. The impedance response should then be analyzed using physically justified equivalent-circuit models that explicitly distinguish ionic and electronic resistive pathways and interfacial polarization, rather than reporting a single bulk resistance. Mixed-conducting hydrogel studies have, for example, resolved ionic and electronic resistances through parallel resistive/constant-phase-element models, providing separate estimates of σ_ion_ and σ_e_ [69,70]. These assignments should preferably be independently validated using DC polarization with ion-blocking electrodes, or an appropriately adapted Hebb–Wagner-type configuration, in which ionic redistribution progressively suppresses the ionic current and the steady-state residual current is attributed predominantly to electronic transport [71]. Because electrode polarization and parasitic electrochemical reactions can produce erroneous partial conductivities, polarization experiments should be verified using low-bias conditions and, when feasible, four-terminal configurations or independent EIS measurements [71]. Reporting σ_total_ alone should therefore be considered insufficient for hydrogels intended to operate as mixed ionic–electronic interfaces.

This distinction is important because increasing water content can improve ionic conductivity while decreasing electronic connectivity. Swelling separates conductive particles and may move a nanocomposite toward or away from its percolation threshold. Conversely, compression may decrease interparticle distances and increase electronic transport. Therefore, conductivity measured in a freshly prepared undeformed specimen does not necessarily describe the electrical state of the material after hours or days of swelling, degradation, and repetitive tissue movement.

Importantly, conductivity measurements during swelling should be referenced to the instantaneous hydrated geometry rather than to the dimensions of the as-prepared hydrogel. At defined hydration times or swelling ratios, specimen thickness, electrode separation and cross-sectional area should therefore be remeasured and used to calculate conductivity. Measurements should ideally include the initial hydrated state, intermediate swelling states and equilibrium swelling in a physiologically relevant electrolyte at controlled temperature. At each state, σ_ion_, σ_e_ and σ_total_ should be reported together with water uptake or polymer volume fraction. This approach is necessary because swelling simultaneously changes mobile-ion concentration, ionic mobility, conductive-domain separation and sample geometry, all of which can modify the apparent conductivity through different mechanisms.

The Hep–PDA–rGO/PAM system illustrates the importance of network organization. Conductivity increased from approximately 0.16 S/m for the GO-containing hydrogel to as high as 3.63 S/m after reduction and improved distribution of Hep–PDA–rGO. Microscopy indicated that homogeneous distribution of the conductive phase generated a more integrated electron-transport pathway, demonstrating that reduction state and percolation architecture are more informative than nominal graphene concentration alone [66]. The same conductive phase also influenced mechanical behavior and antioxidative activity, making the system relevant to multidomain hydrogel design.

Conductive polymers introduce an additional redox dependence because their electronic transport is linked to oxidation and doping state. PEDOT-based wound hydrogels are therefore particularly relevant to redox–electrical coupling. In the PDA–Fe–PEDOT nanozyme system, PEDOT provided an electronic pathway capable of transmitting externally applied electrical stimulation, while the polyphenol/Fe environment generated redox-catalytic and antioxidant activity [60]. Similarly, the alginate/tannic acid/PEDOT:PSS platform combined dynamic hydrogel mechanics, phenolic redox activity, and skin-relevant conductivity within one polymeric network [68].

Piezoelectric hydrogels differ fundamentally from passive conducting materials because electrical output is generated by mechanical deformation. SrTiO_3_ nanoparticles were incorporated into an injectable conductive gelatin-derived matrix. Bending generated voltages approaching 1 V and currents of approximately 0.5 nA, directly demonstrating conversion of mechanical deformation into electrical output [56]. Such systems should therefore be characterized not simply by conductivity but by electromechanical conversion efficiency, output stability during cyclic deformation, mechanical input amplitude and frequency, hydrated impedance, and the persistence of electrical output during degradation.

Clinical translation of piezoelectric nanocomposite hydrogels requires a distinction between electromechanical stability and biological persistence. Ceramic piezoelectric phases such as BaTiO_3_ and related titanates can maintain electromechanical activity within a hydrogel while the surrounding polymer network progressively hydrates and degrades; however, degradation of the hydrogel matrix does not necessarily imply degradation or clearance of the embedded inorganic nanoparticles. This distinction is particularly relevant for BaTiO_3_, for which limited physiological biodegradability raises questions regarding particle release, local retention, macrophage uptake, biodistribution, and the possibility of persistent foreign-body or inflammatory responses [72,73]. These effects are expected to depend on nanoparticle size, surface chemistry, aggregation state, dose, matrix confinement, and duration of tissue exposure. Consequently, short-term cytocompatibility and histological evaluation over the typical 7–14-day wound-healing window cannot establish chronic biosafety. Translation will require longer-term studies that simultaneously determine preservation of piezoelectric performance, particle liberation from the degrading network, tissue retention and systemic distribution, and the evolution of local immune responses. Biodegradable piezoelectric polymers and bio-derived piezoelectric materials may reduce concerns associated with persistent inorganic nanophases, although their generally lower electromechanical output introduces an important performance–biodegradability trade-off [72,73].

A sodium alginate/PVA hydrogel containing sodium niobate similarly exploited movement at articulating wound sites to generate a local electrical field while simultaneously producing ROS under photodynamic activation [58,74]. This type of platform illustrates why mechanical, redox, and electrical properties should increasingly be evaluated under the same operating conditions rather than as independent characterization panels.

The most sophisticated recent examples integrate external mechanical energy with both electrical and redox transformations. In the BTO@Au dual-network hydrogel, ultrasound deforms the piezoelectric phase, produces charge separation and a local electric field, and simultaneously drives redox reactions producing ROS. The intensity of the mechanical input changes the relative magnitude of these outputs, allowing high ROS production during antimicrobial treatment and lower ROS plus electrical stimulation during regenerative phases [58,63]. In BT-dNO@Gel, LIFU likewise acts as a common physical input controlling direct current, ROS generation, and NO release [75]. These systems provide much stronger evidence of multidomain coupling than conventional conductive–antioxidant hydrogels.

### 3.5. Hydration, Degradation, and Evolution of the Functional Material State

The physicochemical state of a hydrogel after implantation is not identical to that measured immediately after synthesis. Hydration, protein adsorption, ion exchange, oxidation, enzymatic degradation, repeated deformation, and cellular remodeling continuously alter network structure. This issue is particularly important for the present review because each of these processes can affect mechanical, redox, and electrical behavior simultaneously.

Water uptake decreases polymer volume fraction and generally increases the distance between polymer chains. Mechanically, this can decrease modulus and alter relaxation times. Chemically, it increases diffusion of oxygen, ROS, ions, enzymes, and metabolites toward reactive groups. Electrically, hydration usually facilitates ionic conduction but may disrupt electronically conducting percolation networks by increasing the spacing between nanofillers. Thus, reporting equilibrium swelling without measuring associated changes in mechanics or conductivity misses an important component of structure–property evolution.

Strategies that actively suppress water-induced dimensional changes provide an additional route to preserving hydrogel function under wet physiological conditions. An intrinsically nonswellable multifunctional hydrogel based on dynamic nanoconfinement, in which the network maintained tissue-like mechanics, high stretchability, self-healing behavior, and electrical conductivity despite prolonged exposure to aqueous environments [76]. In that system, suppression of bulk swelling was critical for preserving the optimized mechanical and electrical properties required for stable bioelectronic interfacing. A complementary strategy was reported by Huang et al. [77], who applied an atomic-layer-deposited silicon dioxide (SiO_2_) nanoscale coating to chemically crosslinked poly(vinyl alcohol) hydrogels [77]. The SiO_2_ layer limited volumetric swelling and preserved mechanical and optical stability during accelerated aqueous incubation compared with uncoated PVA hydrogels. Together, these approaches illustrate two distinct stabilization principles: controlling water uptake intrinsically through network architecture and restricting water-driven dimensional changes extrinsically through nanoscale inorganic barrier coatings.

For regenerative hydrogels, however, minimizing swelling should not be treated as an isolated objective, because excessive restriction of hydration may also alter ion mobility, molecular diffusion, stress relaxation, degradation, and cell–material transport. Wet-state stabilization strategies should therefore be evaluated by their ability to preserve the intended mechanical, redox, and electrical functions rather than by dimensional stability alone.

Degradation has similarly multidimensional consequences. Cleavage of crosslinks reduces the number of elastically active chains, increases mesh dimensions, accelerates transport, and usually enhances stress relaxation. In a conductive composite, the same process may disconnect conductive domains and increase impedance. In a redox-responsive system, oxidative degradation may additionally consume functional groups or release active molecules. Consequently, percentage mass loss should not be treated as an isolated endpoint but as a process that shifts the material through different mechanical, chemical, and electrical states.

An additional translational consideration arises when degradation of the polymer network exposes or releases a persistent piezoelectric nanophase. In this situation, mass loss of the hydrogel is not an adequate surrogate for complete material biodegradation. As polymer connectivity decreases, previously immobilized ceramic nanoparticles may become more accessible to extracellular fluids, phagocytic cells, or systemic transport. The biological consequence therefore depends not only on the degradation rate of the matrix but also on whether the piezoelectric phase dissolves, fragments, remains locally retained, or is redistributed after release. Persistent nanoparticle uptake by macrophages may alter the duration and phenotype of the foreign-body response, whereas systemic redistribution introduces additional requirements for evaluating organ accumulation and clearance [72,73]. Longitudinal assessment should therefore combine hydrogel mass loss with particle-specific quantification, local histopathology, macrophage and inflammatory markers, and evaluation of major organs over time. Importantly, these measurements should be correlated with the residual piezoelectric response because a formulation that remains electrically active but leaves a persistent inorganic burden after the therapeutic window may be less clinically favorable than a system exhibiting controlled loss of both structural and electromechanical function.

This perspective is supported by recent mechanically adaptive hydrogels intended for diabetic wounds. Ren et al. [75]. developed a gelatin/PVA/grape-seed-extract/quaternary-chitosan system specifically combining tissue-compatible mechanical adaptability, antioxidant/anti-inflammatory functionality, and bioelectronic conductivity for wound repair and epidermal electrophysiological monitoring [58]. The significance of such systems lies not simply in the number of functions incorporated but in the possibility of maintaining bioelectronic performance while the hydrogel experiences large deformation and a chemically aggressive wound environment.

Recent double-network and piezoelectric systems push this principle further by deliberately allowing the functional state of the hydrogel to change during treatment. The BTO@Au system combines temperature-sensitive network softening and contraction with mechanically induced electric fields and ROS generation [58]. In the BT-dNO@Gel platform, the material maintained adhesion and self-healing behavior while degrading almost completely in physiological media within approximately 10 days; during this period, LIFU could repeatedly activate electrical, redox, and NO-related functions [63]. These examples demonstrate that the relevant design variable is not a static set of properties at t = 0, but the trajectory of the material state during treatment.

Accordingly, hydrogel design for skin regeneration should increasingly be evaluated through correlated measurements. Mechanical properties should be measured before and after hydration, oxidative exposure, electrical stimulation, and degradation; electrical impedance and conductivity should be evaluated under cyclic strain and during swelling; and redox activity should be examined before and after mechanical or electrical activation. Only these cross-domain experiments can distinguish true coupling from the simultaneous presence of unrelated functions.

**Figure 3:**
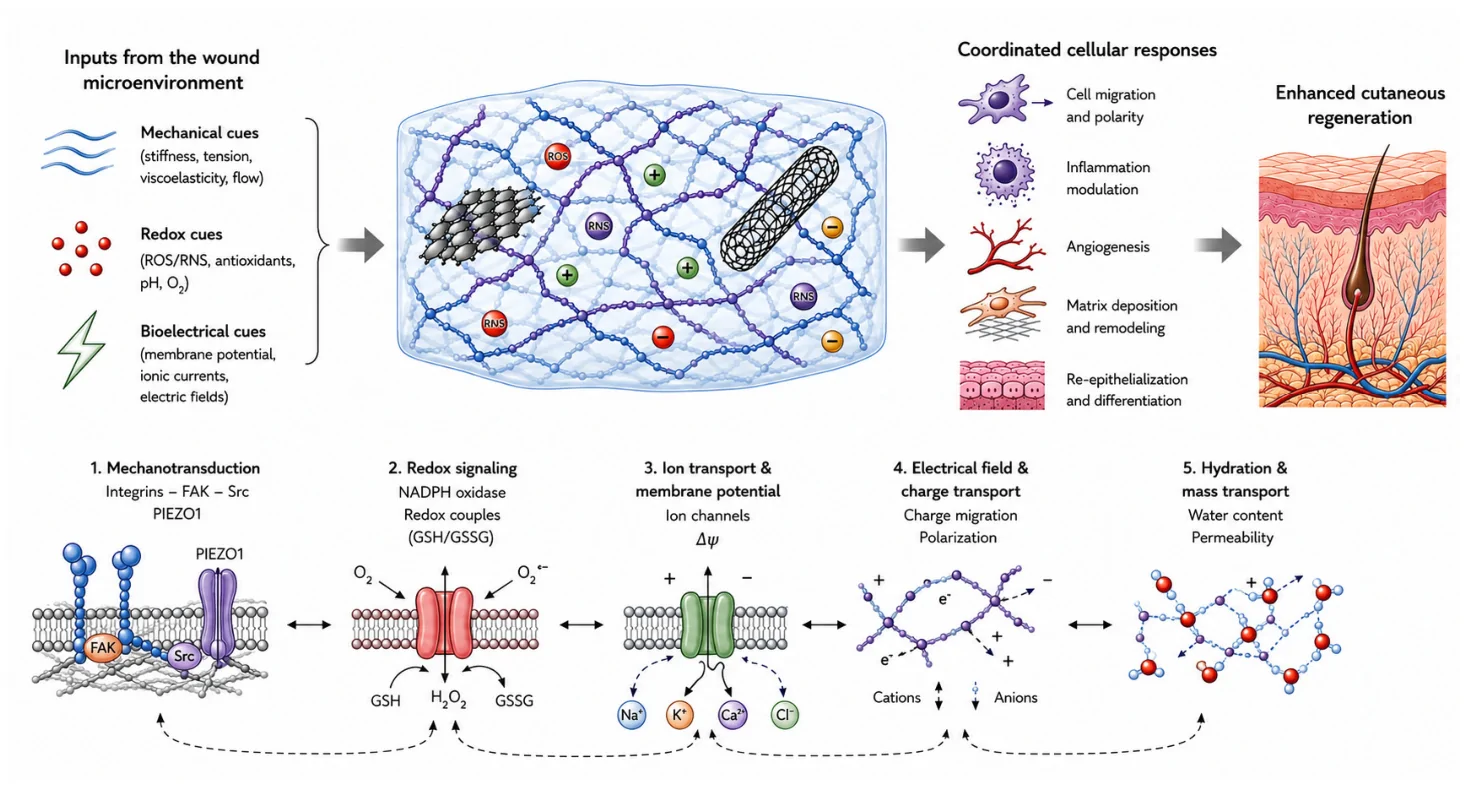
Conceptual design framework linking hydrogel network architecture with mechanochemical, redox, and bioelectrical transduction. The arrows represent evidence-informed structure–property interdependencies synthesized in this review and should not be interpreted as universally established causal pathways. Representative experimental evidence supporting individual relationships is discussed in Section 3.

Figure 3 summarizes the resulting structure–property framework proposed in this review. Unlike the molecular pathways depicted in Figure 1 and Figure 2, the arrows in Figure 3 represent conceptual, evidence-informed interdependencies between network architecture, hydration, mechanics, redox chemistry, electrical transport, and degradation rather than universally established causal relationships. Representative experimental support for individual relationships is provided by the hydrogel systems discussed throughout Section 3.

The recent systems summarized in Table 5 were therefore selected using a more restrictive criterion than conventional multifunctionality: each material contains experimentally assessed mechanical, redox, and electrical components relevant to cutaneous repair. Importantly, the table also distinguishes the presence of all three domains from demonstrated multidomain coupling. This distinction is essential because most current hydrogels still occupy an intermediate stage in which three functionalities coexist, while only a smaller group demonstrates causal conversion of one signal into another.

## 4. Cross-Domain Coupling in Hydrogel Networks: Mechanisms, Quantification, and Design Criteria

The development of multifunctional hydrogels has enabled mechanical reinforcement, redox regulation, electrical conduction, drug delivery, antimicrobial activity, and tissue adhesion to be incorporated within individual wound dressings. However, increasing the number of functions incorporated into a formulation does not necessarily increase the degree of physicochemical integration of the material. The central question is therefore no longer whether a hydrogel exhibits mechanical, redox, and electrical properties simultaneously, but whether energy, charge, chemical potential, or structural information can be transferred between these domains in a controlled and experimentally measurable manner.

For the purposes of this review, cross-domain coupling is defined at the material level as a condition in which a controlled perturbation of one physicochemical domain generates a reproducible change in another domain while composition and confounding variables are held constant. This definition is intentionally more restrictive than the terms *multifunctionality* or *synergy*. For example, a conductive antioxidant hydrogel displaying a modulus of 20 kPa, 80% radical scavenging, and a conductivity of 1 S/m demonstrates three simultaneous properties, but these independent measurements do not establish that a change in redox state alters conductivity or that deformation changes redox activity. By contrast, a piezoelectric hydrogel in which an imposed strain produces a measurable potential fulfills a direct mechanical-to-electrical cross-response, while a piezocatalytic hydrogel in which mechanical excitation modifies ROS production provides direct mechanical-to-redox evidence.

This distinction can be formalized using a response-function approach. For small perturbations around a defined material state, changes in mechanical (M), redox (R), and electrical (E) outputs may be represented conceptually in Equation (1).

(1) $$\left[\begin{array}{c} \Delta M\\ \Delta R\\ \Delta E \end{array}\right]=\left[\begin{array}{ccc} K_{MM} & K_{MR} & K_{ME}\\ K_{RM} & K_{RR} & K_{RE}\\ K_{EM} & K_{ER} & K_{EE} \end{array}\right]\left[\begin{array}{c} X_{M}\\ X_{R}\\ X_{E} \end{array}\right];$$

where the diagonal terms describe conventional responses within the same domain, whereas the off-diagonal coefficients describe cross-domain responses. Thus, K_EM_ represents an electrical response to mechanical input, K_RM_ a redox response to mechanical input, and K_ER_ an electrical response to a controlled redox perturbation. This representation is conceptually related to linear-response formulations used in nonequilibrium thermodynamics, although strict Onsager reciprocity should not be assumed for wound hydrogels because many operate under large deformation, irreversible degradation, ultrasound, photothermal activation, chemical reactions, and other far-from-equilibrium conditions [79]. This framework provides a useful distinction among coexistence, structural integration, directional transduction, and reciprocal coupling, which represent progressively stronger levels of experimental evidence.

### 4.1. Levels of Functional Integration in Hydrogel Networks

At the lowest level, property coexistence describes a hydrogel in which mechanical, redox, and electrical characteristics are independently measurable but are generated by distinct components or mechanisms. Many conductive antioxidant wound dressings belong to this category. A polymer network may determine stiffness, a polyphenol may provide radical-scavenging activity, and graphene or PEDOT may provide conductivity, yet no experiment establishes a functional dependence among them.

A higher level is structural integration, in which a common molecular or nanoscale component contributes to two or more properties. The Hep–PDA–rGO/PAM hydrogel is illustrative: the graphenic/PDA phase participates in mechanical reinforcement, antioxidant activity, electrical conduction, and deformation-dependent resistance. Nevertheless, because redox state was not independently perturbed while conductivity or mechanics were monitored, the system provides stronger evidence for structural integration than for complete three-way coupling [66].

Directional cross-domain transduction requires stronger evidence. Here, a defined input in one domain generates an output in another. Mechanical deformation producing voltage in a piezoelectric hydrogel is the clearest example. Similarly, ultrasound-dependent ROS generation from a piezoelectric catalytic phase constitutes mechanical-to-redox transduction. Importantly, directionality matters: evidence that deformation changes conductivity does not automatically establish that electrical stimulation modifies mechanics.

The strongest level would be reciprocal or feedback coupling, in which a first cross-domain transformation modifies the material state sufficiently to alter subsequent responses. For example, mechanical deformation could produce ROS; those ROS could oxidize dynamic crosslinks and change stress relaxation; the altered mechanics could subsequently modify piezoelectric stress transfer and change electrical output during the next deformation cycle. Although physically plausible, complete feedback loops of this type have not yet been convincingly demonstrated in cutaneous hydrogel systems. These progressively stronger levels of functional integration and their minimum experimental requirements are summarized in Table 6.

This hierarchy is particularly useful because it avoids assigning the same mechanistic weight to materials that merely contain multiple functional ingredients and materials in which physical energy conversion has been experimentally demonstrated.

### 4.2. Spatial and Interfacial Requirements for Cross-Domain Coupling

Cross-domain coupling occurs locally at interfaces and therefore depends strongly on the spatial organization of the network. Mechanical stress is transmitted through polymer chains and junctions, electronic current through connected conductive phases, ionic current through hydrated channels, and redox reactions through accessible molecular or catalytic sites. Effective coupling consequently requires these pathways to intersect over relevant length scales.

Conductive nanocomposites provide a clear example. Below the effective percolation threshold, conductive particles remain electrically isolated even if their intrinsic conductivity is high. Above this threshold, continuous or tunneling-connected pathways can emerge. Mechanical deformation then changes particle separation, orientation, contact area, and tunneling distances, producing an electrical response. The result is not determined solely by nanomaterial concentration but by filler dispersion, aspect ratio, polymer–filler interactions, swelling, and network topology.

This principle was experimentally evident in the Hep–PDA–rGO/PAM system, in which chemical reduction and improved dispersion of rGO substantially increased electrical conduction while the graphenic phase simultaneously reinforced the hydrogel. Conductivity reached 3.63 S m^−1^, and deformation caused measurable resistance changes, directly demonstrating that the mechanical configuration of the percolating network influences electrical transport [66]. The significance of this result for coupling is greater than the conductivity value itself: deformation is able to reconfigure the charge-transport pathway.

A different interfacial mechanism occurs in conducting-polymer/polyphenol systems. Assembled PEDOT on polydopamine-modified silk microfibers embedded within an alginate/gelatin hydrogel. PEDOT-containing fibers increased compressive strength from approximately 82 to 100 kPa and produced conductivity of 13.12 ± 1.66 S/m. More importantly, XPS showed an increase in C–O and decrease in C=O species after PEDOT assembly, which the authors attributed to electron transfer from PEDOT toward PDA and consequent shift in the catechol/quinone balance.

This example is particularly relevant because electrical transport and redox chemistry occur at the same polymeric interface. The redox-active PDA is not spatially separated from the PEDOT conductor; instead, the electronic phase participates directly in controlling the chemical state of the polyphenol. Such architectures provide stronger evidence for redox–electrical coupling than systems in which an antioxidant compound and conductive filler are independently dispersed.

Hydration introduces an additional interfacial constraint. Water facilitates ionic transport and accessibility of ROS to reactive sites but can also screen electrostatic interactions, increase distances between electronically conducting domains, and alter stress transfer between the polymer and embedded nanomaterials. Consequently, coupling measured in dry or partially hydrated states cannot necessarily be extrapolated to physiological conditions. This is particularly important for conducting polymer systems because conductivity, morphology, ionic penetration, and doping state can all change in aqueous environments. Recent work on highly conductive PEDOT-based hydrogels has demonstrated that aqueous processing and molecular dispersion can substantially alter conductivity while maintaining soft mechanical behavior, reinforcing the importance of the hydrated microstructure rather than conductive-polymer content alone.

For coupling-oriented design, therefore, the relevant structural descriptor is not merely the concentration of a functional component but its effective connectivity with load-bearing, redox-active, and charge-transporting domains.

### 4.3. Mechanical-to-Electrical and Mechanical-to-Redox Energy Transduction

Mechanical input represents an especially attractive trigger for wound hydrogels because deformation is intrinsically available through body motion, tissue contraction, pressure, or externally applied ultrasound. Mechanically responsive systems can exploit this energy either by reorganizing conductive pathways or through direct electromechanical conversion.

In piezoelectric composites, stress transferred from the polymer network to an electroactive phase changes polarization and generates a surface potential. However, the effective response of a hydrogel cannot be inferred from the intrinsic piezoelectric coefficient of the filler alone. The magnitude of the resulting signal depends on stress-transfer efficiency, filler concentration and orientation, dielectric properties, interfacial adhesion, hydration, charge screening, electrical leakage, and the continuity of conductive pathways.

An electromechanical conversion was demonstrated in an injectable gelatin-derived conductive hydrogel containing SrTiO_3_ nanoparticles. Mechanical bending generated electrical output approaching approximately 1 V and 0.5 nA, demonstrating a direct input–output relationship between deformation and electrical activity rather than passive conductivity alone [56].

Recent wound hydrogels extend this mechanism into mechanoredox transduction by exploiting piezocatalysis. Mechanical deformation of a semiconductor piezoelectric phase induces separation of electrons and holes. When these charge carriers reach the material–water interface, they can participate in reduction and oxidation reactions involving dissolved O_2_, H_2_O, and other species, producing reactive intermediates. The mechanical input therefore becomes a determinant of chemical reaction rate.

The BTO@Au hydrogel provides one of the most complete demonstrations of this mechanism in a cutaneous model. Au-modified BaTiO_3_ formed metal/semiconductor interfaces that promoted charge separation, while ultrasound deformation generated piezoelectric polarization. Increasing ultrasound power from 0.3 to 1.5 W/cm progressively increased both singlet-oxygen and hydroxyl-radical generation, demonstrating a controllable mechanical-input/redox-output relationship [58].

The same study also demonstrates why coupling should not be interpreted as simple maximization of output. High ultrasound power was used to produce bactericidal ROS, whereas lower power generated less ROS and a local microelectric field associated with more favorable migration and angiogenic responses. At 0.5 W/cm, fibroblast migration reached an approximately 88% closure ratio in the reported in vitro assay. Thus, the functional state of the material is dependent on input intensity, and different operating regimes can favor different outputs from the same material.

The LIFU-responsive BT-dNO@Gel system reported by Li et al. [63]. further illustrates multidomain energy conversion. Mechanical energy supplied by low-intensity focused ultrasound is converted by BaTiO_3_ into microcurrents and ROS, while the same stimulation induces local NO release. The authors experimentally varied LIFU conditions and demonstrated that ROS generation depended on power and exposure conditions. The system therefore exhibits a common mechanical input controlling electrical and chemical outputs [63].

These systems establish a key principle for next-generation gels: the coupling variable is the transfer function between input and output, rather than the maximum magnitude of either property.

### 4.4. Redox–Electrical Coupling and Charge-Storage Processes

Redox–electrical coupling is less mature experimentally than mechanoelectrical coupling, despite being particularly interesting from the perspective of hydrogel chemistry. Electronic transport and redox reactions both involve charge transfer, and conducting polymers, graphenic phases, metal–phenolic complexes, and quinone-containing networks can therefore provide molecular interfaces where the two phenomena are intrinsically connected.

Conducting polymers are especially relevant because their electronic transport depends on oxidation and doping state. PEDOT, polypyrrole (PPy), and polyaniline (PANI) contain conjugated backbones in which oxidation or reduction changes the density and mobility of charge carriers. When such polymers are interfaced with redox-active groups, electron transfer can modify the chemical state of the latter while simultaneously influencing the electronic structure of the conductive phase.

The PEDOT–PDA system provides unusually direct evidence. PEDOT incorporation changed the PDA catechol/quinone ratio detected by XPS, and reductive H_2_S further increased the fraction of catechol-associated C–O groups. The same PEDOT network supplied high conductivity that remained stable under repeated compression and after implantation [80]. While the model is periodontal rather than cutaneous, it provides a strong material-level example of how conductive polymer chemistry can influence redox state.

Recent alginate-based redox-capacitive hydrogels provide another relevant direction. A metal-free alginate/rGO/proanthocyanidin system specifically designed to integrate phenolic redox chemistry with graphenic conductivity and to evaluate charge-storage behavior by impedance and electrochemical analysis. Such systems are conceptually important because the relevant electrical descriptor is no longer conductivity alone; charge-transfer resistance, interfacial capacitance, pseudocapacitive storage, and redox accessibility become part of the functional state of the hydrogel.

This distinction suggests that electrochemical impedance spectroscopy (EIS) may be particularly valuable for coupled hydrogels. Changes in charge-transfer resistance (Rct), capacitive or constant-phase behavior, ionic diffusion, and low-frequency impedance can potentially reveal alterations of redox-accessible interfaces that conventional two-point conductivity measurements cannot resolve. However, electrochemical models must be interpreted cautiously because changes in swelling, electrolyte concentration, electrode contact, or geometry can produce impedance changes unrelated to intrinsic redox transformations.

The PDA–Fe–PEDOT conductive nanozyme provides a wound-specific example in which redox catalytic and conductive functions are colocated. The material preserved antioxidant function during electrical stimulation and combined H_2_O_2_-dependent catalytic behavior with PEDOT-mediated electrical transmission during diabetic wound treatment [60]. Nevertheless, direct plots of conductivity as a function of controlled oxidation state remain uncommon, highlighting an important experimental gap.

A rigorous demonstration of material-level redox–electrical coupling would ideally vary a controlled redox coordinate—such as potential, H_2_O_2_ concentration, reduced glutathione (GSH) concentration, catechol/quinone ratio, or conducting-polymer oxidation state—and simultaneously determine conductivity, impedance, carrier transport, or charge-storage capacity. Without such experiments, redox and electrical measurements remain largely correlative.

Beyond mechanistic characterization, redox–electrical coupling is particularly relevant to the development of wound interfaces that combine biochemical sensing with therapeutic actuation. Changes in H_2_O_2_ concentration, oxidation state, or other wound-associated redox variables can modify interfacial charge transfer, impedance, capacitance, or conductivity and may therefore be converted into electrically readable indicators of wound state. Conversely, an electrical input can modify conducting-polymer oxidation, catalytic activity, ROS generation, or electrically controlled therapeutic delivery. This bidirectional relationship creates a potential sensing–actuation bridge in which local redox chemistry provides information about the wound microenvironment and the electrical domain provides both a measurable output and a route for therapeutic intervention. Recent conductive redox hydrogels already combine electrical stimulation with sustained ROS regulation, although direct autonomous feedback remains to be demonstrated [60].

Representative experimental systems spanning different levels of cross-domain coupling are compared in Table 7.

### 4.5. State Dependence, Hysteresis, and Temporal Evolution of Coupling

A cross-domain coefficient measured immediately after fabrication should not be regarded as an immutable material constant. Hydrogel coupling depends on an evolving state defined by hydration, ion exchange, deformation history, redox exposure, degradation, temperature, and protein adsorption. Consequently, the cross-response should more realistically be written as Equation (2).

(2) $$K_{ij}=K_{ij}(t,\lambda ,\phi_{w},\;\xi ,\Psi ,T,pH)$$

where t represents time, λ deformation, ϕ_w_ water fraction, ξ network integrity or degradation state, Ψ redox/electrochemical state, and T temperature. This expression is conceptual rather than a universal constitutive equation, but it emphasizes that coupling is state dependent.

This concept is especially important near percolation thresholds. Swelling can increase the separation between graphenic sheets or conductive-polymer domains and thereby alter electronic transport, while simultaneously increasing ionic mobility. Mechanical compression may produce the opposite effect by bringing conductive domains into closer contact. Hence, an observed change in conductivity after deformation may originate from electronic percolation, ionic concentration changes, or both. Mechanistic interpretation therefore requires separating ionic from electronic contributions.

Under mechanically deformed conditions, this separation should be performed in situ rather than inferred from resistance measurements obtained before and after loading. EIS should be acquired at predefined tensile or compressive strains while maintaining hydration, temperature, electrode contact, and electrolyte composition constant. Sample dimensions should be recorded at each deformation state so that resistance changes caused purely by variations in current-path length or cross-sectional area can be distinguished from changes in intrinsic conductivity. Selected deformation states should additionally be evaluated by DC polarization to determine whether strain preferentially alters σ_ion_ or σ_e_. Cyclic loading should include loading–unloading measurements to quantify electrical hysteresis and determine whether conductive pathways recover after deformation. An ionic-conducting control lacking the electronic phase and, where applicable, a composition-matched electronically conductive control are particularly valuable for assigning the origin of the strain-dependent response.

Hysteresis provides another important but underexplored descriptor. If a hydrogel is stretched and released, its electrical response may not retrace the same path because conductive domains, dynamic bonds, or fluid distributions may rearrange irreversibly or with finite relaxation times. Similarly, oxidation of catechol or conducting-polymer groups can alter network interactions and produce chemical memory. A coupled hydrogel may therefore exhibit a history-dependent response even when the external stimulus returns to its initial value.

State dependence is experimentally evident in the BTO@Au system. Increasing BTO@Au loading increased the compressive stress at 60% strain from approximately 0.014 to 0.116 MPa but simultaneously decreased fracture strain from approximately 95% to 67%. The same compositional increase reduced thermoresponsive surface contraction, demonstrating that maximizing the electroactive phase imposes a mechanical penalty on chain mobility [58]. This is an important example of a cross-property trade-off that would be obscured if conductivity, piezoelectric response, and mechanics were optimized independently.

The same study showed an additional feedback-like behavior at the thermal level: contraction increased the local concentration of BTO@Au per unit volume and enhanced photothermal conversion, demonstrating that network deformation can alter the effective concentration and therefore the functional output of the embedded phase. Although this is not yet a complete mechanoredox–electrical feedback loop, it illustrates how physical rearrangement of the network can modify subsequent energy conversion.

Likewise, the Ag_2_Se@GelMA thermoelectric system was explicitly evaluated over extended hydration periods, with Seebeck coefficient and electrical conductivity followed at several time points up to 15 days, emphasizing the need to establish whether self-powered electrical function survives the changing hydrated state of the dressing [59].

Accordingly, coupling studies should evolve from single-point measurements toward state maps in which mechanical, redox, and electrical cross-responses are determined as functions of time and relevant environmental conditions.

### 4.6. Quantitative Metrics for Cross-Domain Coupling

A major limitation of the current literature is that coupling is generally described qualitatively. Terms such as synergistic, integrated, and coupled are frequently applied without quantifying the dependence of one property on another. Establishing quantitative descriptors would substantially improve comparison between hydrogel systems.

For a controlled change in an input, a differential cross-response can be expressed as Equation (3).

(3) $$x_{ij}=\frac{\partial Y_{i}}{\partial X_{j}}$$

Because mechanical, electrical, and redox quantities have different units, comparison among systems may require a normalized sensitivity. For discussion purposes, this review proposes Equation (4).

(4) $$S_{ij}=\frac{\Delta Y_{i}/Y_{i,0}}{\Delta X_{j}/X_{j,0}}$$

where X_j,0_ and Y_i,0_ are reference-state values. This expression is proposed here as a comparative conceptual metric and is not an established standardized coupling coefficient.

For mechanoelectrical systems, established parameters can already approximate such relationships. Gauge factor quantifies resistance sensitivity to strain, while effective piezoelectric coefficients relate mechanical stress or strain to generated electrical displacement. However, reporting voltage alone is insufficient because output depends on specimen dimensions, electrode configuration, loading amplitude, frequency, and impedance. Mechanical input should therefore be reported together with charge, current density, power density, or effective energy-conversion efficiency.

For mechanoredox systems, analogous metrics are less standardized. Relevant outputs could include ROS generation rate per unit stress, strain, acoustic power, or mechanical energy. The BTO@Au study provides an example because radical generation increased systematically with ultrasound power density, whereas the BT-dNO@Gel work similarly evaluated ROS output as a function of LIFU parameters [58,63].

For redox–electrical coupling, particularly useful quantities would include Rct, impedance magnitude and phase, capacitance or constant-phase parameters, current response, and conductivity as functions of redox potential or chemically determined oxidation state. Conducting-polymer systems offer a strong platform because the oxidation/doping state can potentially be correlated directly with charge transport.

Finally, multidomain systems should ideally be evaluated by simultaneous rather than sequential measurements. A hydrogel exposed to mechanical stimulation could be monitored concurrently for voltage/current, impedance, ROS production, and rheological changes. Such experiments would reveal whether responses arise from the same event or only coexist under nominally similar conditions.

### 4.7. Causality, Controls, and Experimental Validation

Demonstrating cross-domain coupling requires more stringent controls than demonstrating multifunctionality. A change in one output following stimulation does not necessarily identify the physical pathway responsible for that change.

Ultrasound is a particularly instructive example because it can simultaneously induce deformation, heating, fluid motion, cavitation, and piezoelectric polarization. Therefore, enhanced ROS generation in an ultrasound-responsive hydrogel cannot automatically be assigned to piezocatalysis. Appropriate controls should include the hydrogel without the piezoelectric phase, piezoelectric particles without ultrasound, ultrasound-only controls, and thermal controls reproducing the measured temperature rise. Luo et al. explicitly considered thermal and cavitation-related effects and used experimental controls to separate these contributions in their BTO@Au system.

The study similarly used control groups including Gel, barium titanate-containing hydrogel (BT@Gel), BT@Gel + LIFU, and BT-dNO@Gel + LIFU in diabetic wound models, allowing progressive contributions of the network, piezoelectric component, mechanical stimulus, and NO-generating functionality to be distinguished [63].

Redox–electrical experiments require equally careful controls. Oxidation can alter swelling, ionic strength, pH, and polymer charge density. Any of these factors can change impedance independently of electronic redox coupling. Therefore, measurements should distinguish ionic from electronic transport and should ideally use spectroscopic or electrochemical confirmation of oxidation state alongside electrical measurements.

Mechanical cross-responses introduce further confounders. An apparent reduction in conductivity during stretching may result from percolation disruption, water redistribution, electrode delamination, or geometric changes in sample dimensions. Four-point measurements, appropriate geometric corrections, strain-controlled impedance measurements, and repeated cycling are therefore preferable when feasible.

The most convincing experimental design combines controlled perturbation, direct measurement of cross-response, mechanistic controls, and reversibility testing. Biological outcomes should then be used as downstream validation rather than as the sole evidence that physicochemical coupling occurred. Recommended controls, material-level readouts, and criteria for demonstrating each coupling mode are summarized in Table 8.

For piezoelectric nanocomposite hydrogels, these validation criteria should extend beyond the period required to demonstrate wound closure. Chronic material safety requires longitudinal evaluation of nanoparticle retention or clearance after degradation of the carrier network, together with local foreign-body response, macrophage phenotype, inflammatory signaling, systemic biodistribution, and major-organ histopathology. Repeated mechanical or ultrasonic activation should also be considered because chronic stimulation may alter both particle release and the surrounding inflammatory microenvironment. Ideally, these biological measurements should be performed in parallel with residual piezoelectric output to determine whether loss of therapeutic function and elimination of the material occur within compatible time scales.

### 4.8. From Feed-Forward Transduction to Adaptive Material Feedback

The strongest recent systems remain predominantly feed-forward devices. In piezoelectric wound hydrogels, mechanical or ultrasonic energy generates polarization, and polarization subsequently produces electrical fields, redox reactions, or both. The BTO@Au and BT-dNO platforms represent advanced examples of this architecture, but the dominant sequence remains mechanical input → electrical/redox output [58,63].

Mechanoredox coupling offers a particularly attractive route toward autonomous or minimally powered wound therapy because mechanical energy is inherently available from tissue deformation, body motion, or externally delivered ultrasound. Rather than using mechanical stimulation solely to deform the dressing, this energy can be converted into temporally localized redox outputs, including controlled ROS production or stimulus-dependent release of redox-active mediators. Such behavior could enable stage-dependent treatment in which a stronger mechanically triggered oxidative response is used transiently for antimicrobial action, followed by lower redox activity during the regenerative phase. The LIFU-responsive BT-dNO@Gel system illustrates this possibility by using a common mechanical input to generate piezoelectric current, ROS, and NO release [63]. Future mechanoredox systems could advance further if the generated redox products also modify ROS-sensitive crosslinks, swelling, degradation, or stress transfer to the piezoelectric phase, thereby changing the magnitude of the subsequent mechanical-to-redox response.

Redox–electrical coupling may be even more directly suited to closed-loop wound management because redox-associated biomarkers can be converted into electrical signals that are compatible with wearable monitoring and subsequently linked to therapeutic actuation. In this architecture, elevated oxidative or inflammatory activity could alter an electrochemical signal, which would then initiate or adjust electrical stimulation, drug release, catalytic ROS regulation, or another therapeutic output. Recent Pd@Au nanoframe hydrogels illustrate an integrated monitoring–therapy strategy by combining wound-state sensing with externally controlled treatment [8], while a more recent electroactive hydrogel integrates electrochemical H_2_O_2_ monitoring with ROS-responsive therapeutic release [82]. These systems indicate a transition from multifunctional dressings toward feedback-responsive platforms in which biochemical information generated by the wound can influence subsequent treatment.

For the purposes of regenerative hydrogel design, however, integration of sensing and therapy should not automatically be considered a complete closed-loop system. Strong evidence for closed-loop operation would require at least four sequential elements: (i) measurement of a wound-associated state variable; (ii) conversion of that information into a defined material or device response; (iii) therapeutic actuation that modifies the wound state; and (iv) subsequent remeasurement that alters the magnitude, duration, or type of the next therapeutic response. Thus, the defining feature is not simply simultaneous sensing and treatment but state-dependent feedback between successive therapeutic cycles.

Several chemical mechanisms could theoretically enable this behavior. ROS-sensitive crosslinks could change network connectivity following piezocatalytic ROS generation; catechol oxidation could change adhesion and network association; conductive-polymer oxidation could change electrical transport; redox-induced swelling could modify filler spacing; and degradation could alter stress transfer to piezoelectric particles. These mechanisms would transform a linear transducer into a history-dependent material system.

Such behavior would be especially relevant to cutaneous regeneration because the desired material response is unlikely to remain constant throughout treatment. Rather than delivering fixed stimulation, a responsive network could progressively modify its transduction behavior as oxidative stress falls, tissue mechanics recover, or the hydrogel degrades.

However, the experimental burden required to prove this level of coupling is substantial. At minimum, the initial stimulus must alter a second physicochemical state; that second state must be experimentally shown to modify a third property or subsequent response; and the sequence should ideally be reversible or reproducibly history-dependent. Current cutaneous hydrogel studies have not yet provided comprehensive evidence satisfying all of these criteria.

This gap provides a more precise future target than the generic development of increasingly “multifunctional” wound dressings.

### 4.9. Design Implications for Regenerative Hydrogels

The preceding analysis indicates that the design of mechanically, redox-, and electrically active hydrogels should move away from the independent maximization of individual properties and toward the controlled regulation of cross-domain responses. Increasing filler concentration, antioxidant loading, stiffness, or conductivity does not necessarily improve regenerative performance because these variables are physically and chemically interconnected and frequently exhibit competing effects. An increase in the concentration of an electroactive nanophase, for example, may improve charge generation or conductivity while simultaneously restricting polymer-chain mobility, reducing extensibility, modifying swelling, or altering degradation. The BTO@Au system illustrates this trade-off particularly well: increasing BTO@Au content enhanced compressive resistance and tensile strength but reduced fracture strain and thermoresponsive contraction, demonstrating that the formulation that maximizes piezoelectric or catalytic activity does not necessarily provide the most favorable mechanical adaptability [58].

A similar principle applies to redox–electrical systems. Conductive components should not be regarded simply as inert pathways for charge transport because their oxidation state, interfacial chemistry, and interactions with redox-active groups can modify several physicochemical properties simultaneously. In PEDOT–PDA-based systems, for example, the conductive phase contributes not only to electrical transport but also to mechanical reinforcement and to the chemical state of catechol/quinone groups through interfacial electron-transfer processes [80]. Such behavior illustrates why the design problem is intrinsically multivariable: changes introduced to improve one domain may alter the operating state of another, and therefore the relevant optimization target is not the maximum value of any single property but the balance between interacting material responses.

From this perspective, the most informative design parameter is the cross-domain sensitivity of the hydrogel under physiologically relevant conditions. A moderately conductive material whose electrical response changes reproducibly with deformation may be more useful than a highly conductive hydrogel that is electrically insensitive to tissue motion. Likewise, controlled and stimulus-dependent ROS production may be more desirable than either constitutively high ROS generation or maximal antioxidant activity, because the biological function of reactive species depends on dose, timing, and spatial distribution. Similarly, a mechanically robust hydrogel may become maladaptive if its stress-relaxation behavior prevents progressive transfer of mechanical load to regenerating tissue. Consequently, the magnitude of a cross-response, its selectivity relative to undesired responses, its temporal stability, and its evolution during hydration and degradation should all be considered during material optimization.

This shift also changes how structure–property relationships should be formulated. Conventional biomaterial development commonly follows a linear sequence in which composition determines a material property and that property is subsequently correlated with a biological outcome. For cross-domain hydrogels, a more appropriate framework considers how network architecture determines a set of transfer functions linking mechanical deformation, redox chemistry, and charge transport; how those relationships evolve as the hydrogel hydrates, deforms, oxidizes, and degrades; and how the resulting material state influences cellular and tissue responses. The relevant relationship is therefore better described as network architecture → cross-domain transduction → evolving physicochemical state → biological response, rather than as a simple composition–property–function sequence.

This framework also implies that hydrogel optimization should increasingly be performed under conditions that reproduce the coupled perturbations encountered in vivo. Mechanical testing performed only immediately after gelation, antioxidant assays conducted in isolated chemical solutions, and conductivity measurements obtained under static conditions provide useful baseline information but cannot establish whether functional coupling persists during wound treatment. More informative experiments would assess electrical response during cyclic deformation, redox activity during mechanical or electrical stimulation, conductivity following controlled oxidation or reduction, and mechanical properties after repeated redox challenges or partial degradation. Such measurements would provide direct evidence that the network maintains or adaptively modifies its cross-domain behavior under physiologically relevant conditions.

The current literature suggests that mechanoelectrical coupling is the most experimentally mature component of this framework because deformation-dependent resistance and piezoelectric voltage or current can be measured directly. Mechanoredox coupling is advancing rapidly through piezocatalytic and mechanically activated redox systems, whereas redox–electrical coupling is increasingly supported by conducting-polymer/polyphenol and redox-capacitive architectures but remains less systematically quantified. The most advanced wound hydrogels now achieve multidomain feed-forward transduction, in which a mechanical or thermal input generates electrical and chemical outputs within the same material. However, truly reciprocal systems in which these outputs subsequently alter network mechanics, redox state, or charge transport and thereby modify the response to future stimuli remain largely unexplored.

Accordingly, the next generation of regenerative hydrogels should not be defined primarily by the number of functionalities incorporated into the formulation, but by the degree to which these functionalities are physically integrated, quantitatively coupled, temporally controllable, and stable under physiological operation. Achieving this objective will require a transition from descriptive multifunctionality toward mechanistically defined structure–property–property–biological relationships, in which the hydrogel is treated as a dynamic soft transduction system rather than as a passive scaffold carrying independent therapeutic functions. Such an approach provides a more rigorous basis for engineering materials capable of adapting to the changing mechanical, oxidative, and electrical conditions that characterize cutaneous regeneration.

## 5. Conclusions

Hydrogels for cutaneous regeneration are evolving from multifunctional materials toward systems capable of integrating mechanical, redox, and bioelectrical signals within a common network architecture. The coexistence of stiffness, antioxidant activity, and conductivity should not be interpreted as functional coupling unless perturbation of one physicochemical domain measurably affects another. Current evidence suggests that mechanoelectrical coupling is comparatively well established, particularly in conductive, piezoelectric, and deformation-responsive systems, whereas mechanoredox and redox–electrical interactions remain less systematically demonstrated. Future studies should therefore prioritize causal validation through controlled perturbations, time-resolved measurements, and simultaneous characterization of mechanical, redox, electrical, and biological responses. Mechanoredox and redox–electrical coupling are particularly promising in this context because they could connect endogenous wound-state changes with sensing, energy transduction, and on-demand therapeutic actuation, providing a mechanistic basis for future adaptive and closed-loop wound interfaces. Overall, a more informative design framework is network architecture → cross-domain transduction → evolving physicochemical state → biological response, which may guide the transition from property coexistence toward directional coupling and adaptive feedback, enabling hydrogels to function as dynamic interfaces that actively interact with the changing wound microenvironment and promote coordinated tissue regeneration.

## Figures and Tables

**Table 2:** Representative primary studies defining redox signaling and redox-homeostasis mechanisms relevant to cutaneous wound healing and skin regeneration.

Model	Redox Signal/Intervention	Molecular Mechanism	Biological Evidence	Implications for Redox-active Hydrogel Design	Evidence Level
Murine dermal wounds; endothelial cells [28]	Endogenous and topical H_2_O_2_; catalase overexpression	H_2_O_2_ → site-specific FAK phosphorylation → angiogenesis	Micromolar H_2_O_2_ was detected in wound fluid. Low-dose H_2_O_2_ supported angiogenesis and closure, whereas high doses impaired healing; catalase overexpression delayed angiogenesis and closure.	Establishes that wound repair requires a redox signaling window, not complete ROS suppression.	A/C/G
Zebrafish epithelial wounds [30]	Tissue injury; DUOX inhibition	DUOX → spatial H_2_O_2_ gradient → leukocyte recruitment	H_2_O_2_ increased within 3 min, peaked at 20 min, and formed a 100–200 μm gradient required for rapid leukocyte recruitment.	Seminal evidence that ROS encode spatial wound information.	NM/G
Zebrafish wounds; leukocyte models [31]	Wound-derived H_2_O_2_	H_2_O_2_ → Lyn Cys466 oxidation → leukocyte chemotaxis	Lyn functioned as a molecular redox sensor necessary for efficient wound-directed leukocyte migration.	Demonstrates conversion of an extracellular ROS gradient into a specific intracellular signaling response.	NM/C/G
C57BL/6 excisional wounds [29]	Topical H_2_O_2_, 10 versus 166 mM	Dose-dependent redox signaling; ERK/p38 and inflammatory remodeling	10 mM H_2_O_2_ enhanced angiogenesis and closure, whereas 166 mM delayed closure, reduced connective tissue, increased MMP-8, and prolonged neutrophil infiltration.	Strong experimental evidence for a non-monotonic redox response.	A
Drosophila epithelial wounds [32]	Injury-induced Ca^2+^ flashes	Ca^2+^ → DUOX → H_2_O_2_ → inflammatory recruitment	Early Ca^2+^ signals activated DUOX-dependent H_2_O_2_ production and coordinated inflammatory-cell recruitment.	Direct evidence of ionic–redox coupling after injury.	NM/G
Zebrafish epidermis; human keratinocytes [37]	Low-level H_2_O_2_ exposure	H_2_O_2_ → EGF, forkhead box O1 (FOXO1), IκB kinase alpha (IKKα) and migration/cytoprotection programs	Comparative transcriptomics identified conserved H_2_O_2_-responsive migration, adhesion, cytoprotective and anti-apoptotic programs.	Shows that low-level redox signaling reorganizes broad regenerative transcriptional programs.	H/C/NM
Human diabetic wound-related samples; diabetic mice [38]	NRF2 deficiency/activation	KEAP1–NRF2 → antioxidant response and redox homeostasis	NRF2 dysfunction aggravated oxidative stress, apoptosis and delayed repair; NRF2 activation improved healing.	Establishes defective endogenous redox regulation as a feature of diabetic wound pathology.	H/A/G
Fibroblasts; db/db diabetic wounds [34]	Topical Keap1 small interfering RNA (siRNA)	Keap1 ↓ → NRF2 nuclear translocation → NAD(P)H quinone dehydrogenase 1 (NQO1)/manganese superoxide dismutase (MnSOD) ↑ → ROS ↓	Keap1 silencing reduced wound ROS 58%, reduced 8-hydroxy-2′-deoxyguanosine (8-OHdG) 42%, accelerated closure by 7 days, and enhanced granulation and neovascularization.	Strong causal evidence that restoring endogenous redox buffering capacity improves regeneration.	C/A/G
Keratinocyte-specific Nrf2 mouse models; diabetic wounds [35]	Epidermal NRF2 deletion	NRF2 → keratinocyte CCL2 → macrophage EGF → keratinocyte proliferation	Epidermal NRF2 coordinated keratinocyte–macrophage crosstalk; disruption impaired wound repair and resembled aspects of diabetic regeneration failure.	Demonstrates that redox signaling controls intercellular communication, not only antioxidant defenses.	A/C/G
*Nox4*-null mice; mouse/human dermal fibroblasts [36]	Genetic NOX4 deletion	TGF-β–redox signaling independent of obligatory NOX4 activity	NOX4 deletion did not significantly impair wound healing or fibroblast-to-myofibroblast differentiation despite NOX4 induction.	Important negative evidence: ROS-associated expression does not establish causal pathway dependence.	H/C/A/G
Barrier epithelial cells and wound models [39]	Localized DUOX2-derived H_2_O_2_ flashes	PIEZO1 → DUOX2 → H_2_O_2_ → FER/cortactin → actin remodeling	Local H_2_O_2_ flashes controlled lamellipodia, cell connections, migration and retraction-wave-dependent wound closure.	Provides unusually direct evidence for mechanoredox coupling and links ROS to cytoskeletal mechanics.	C/G
Murine dermal wounds; endothelial cells [33]	Endogenous and topical H_2_O_2_; catalase overexpression	H_2_O_2_ → site-specific FAK phosphorylation → angiogenesis	Micromolar H_2_O_2_ was detected in wound fluid. Low-dose H_2_O_2_ supported angiogenesis and closure, whereas high doses impaired healing; catalase overexpression delayed angiogenesis and closure.	Establishes that wound repair requires a redox signaling window, not complete ROS suppression.	A/C/G

Evidence level: H, human tissue/clinical physiology; LA, large-animal model; A, animal model; C, cellular/in vitro; G, genetic or molecular causal perturbation; BM, biomaterial-mediated intervention; NM, non-mammalian mechanistic model.

**Table 3:** Representative primary studies defining endogenous bioelectrical signaling and electrotactic mechanisms relevant to cutaneous wound healing and skin regeneration.

Electrical Signal/Intervention	Molecular Mechanism	Biological Evidence	Implications for Electroactive Hydrogel Design	Evidence Level
Direct-current (DC) electric fields comparable to those occurring in mammalian wounds [41]	Electric-field sensing → cell polarization → cathodal electrotaxis	Keratinocyte migration was essentially random at ≤5 mV/mm but became increasingly cathodal between 10 and 100 mV/mm, demonstrating sensitivity to weak physiological fields.	Hydrogel systems should reproduce physiologically relevant field strengths rather than maximize voltage or conductivity.	H/C
Physiological DC electric field; manipulation of extracellular Ca^2+^ and growth factors [42]	Electric field → extracellular Ca^2+^ + growth-factor signaling → directional migration	Effective keratinocyte galvanotaxis required extracellular Ca^2+^ and growth-factor signaling, demonstrating that electrical guidance depends on the surrounding ionic and biochemical environment.	Electroactive hydrogels should preserve physiological ionic availability and cell–material biochemical signaling; conductivity alone is insufficient.	H/C
Physiological DC electric fields (75–100 mV/mm) [45]	vascular endothelial growth factor receptor (VEGFR) activation → PI3K/Akt and Rho/ROCK → cytoskeletal polarization	Electric fields induced endothelial-cell elongation, orientation and directional migration and stimulated VEGF-associated pro-angiogenic signaling.	Electrical functionality should be related to angiogenic signaling and endothelial responses, not solely to measured conductivity.	C
Physiological electric field (100 mV/mm) [43]	β4-integrin + EGF → Rac1 → directional migration	β4-integrin was required for efficient cathodal migration in the absence of exogenous EGF, demonstrating integration between electrical guidance, cell–ECM adhesion and growth-factor signaling.	Hydrogel ligand presentation and adhesive interactions may influence electrotaxis; electrical and interfacial properties should therefore be co-designed.	H/C/G
Physiological electric fields and experimental modulation of endogenous wound currents [44]	PI3Kγ/PTEN polarization → electrotaxis	PI3Kγ disruption impaired electrically directed migration whereas PTEN loss enhanced it; experimental modulation of endogenous electrical signals altered wound healing in vivo.	Demonstration of bioelectrical functionality should ideally include cell polarization or pathway-level responses, rather than conductivity alone.	A/C/G
Direct measurement and pharmacological modulation of endogenous wound electric fields [48]	Transepithelial ion transport → current of injury → lateral electric field	Immediately after wounding, mouse skin generated fields of 177 ± 14 mV/mm that remained 150–200 mV/mm for several days. Amiloride reduced the field by 64%, whereas prostaglandin E_2_ (PGE_2_) increased it by 82%; endogenous fields were also detected in human wounds.	Hydrogels should be designed to interact with ionic transport and endogenous wound currents, not merely provide an electronically conductive pathway.	H/A
Physiological electric fields of 50–100 mV/mm [49]	Electric field → PI3K/Akt signaling → anodal fibroblast migration	Human dermal fibroblasts migrated slowly toward the anode at 50–100 mV/mm, opposite to the cathodal response of keratinocytes. PI3Kγ deficiency reduced directional migration.	A single electrical field may produce opposing responses in different wound-resident cells; electroactive hydrogels should therefore be evaluated in multicellular systems.	H/C/A/G
Non-invasive measurement of endogenous wound electric fields [40]	Wound-associated ionic current and age-dependent electrical response	Endogenous electric fields were measured around human skin wounds and declined significantly with increasing age, suggesting that physiological bioelectrical signaling varies with regenerative status.	Electrical stimulation may require patient- or wound-dependent tuning rather than a universal field strength.	H
Physiological electric fields; modulation of endogenous wound currents [50]	Electric field → directional epidermal stem-cell migration	Epidermal stem cells responded directionally to physiological electric fields, and modulation of endogenous wound electrical signaling was associated with improved wound repair.	Electroactive hydrogels may support regeneration by preserving or reproducing endogenous guidance cues for progenitor/stem-cell recruitment.	A/C
Electrical stimulation at 50 or 200 mV/mm [46]	Electrical stimulation → fibroblast proliferation/migration → myofibroblast differentiation	Electrical stimulation increased fibroblast proliferation and migration, growth-factor secretion and collagen-gel contraction, but also promoted fibroblast-to-myofibroblast transdifferentiation.	Persistent or excessive electrical stimulation may accelerate repair while simultaneously promoting profibrotic myofibroblast activation; electrical dose and duration must therefore be controlled.	H/C/BM
Exogenous electrical stimulation [51]	Electrical stimulation → SIVA1 apoptosis-inducing factor (SIVA1)–human double minute 2 (HDM2)/tumor suppressor p53 regulation → keratinocyte proliferation	Electrical stimulation enhanced epidermal stratification and keratinocyte proliferation and increased p53–SIVA1-associated signaling in human cutaneous wounds.	Bioelectrical hydrogel performance should be evaluated through defined cellular signaling and proliferative responses, rather than electrical output alone.	H/C
DC electric field (100 mV/mm); purinergic receptor modulation [52]	Electric field → adenosine triphosphate (ATP) release → P2Y purinergic receptor (P2Y) signaling→ galvanotaxis	Exposure to an electric field stimulated extracellular ATP release, while disruption of P2Y signaling impaired directional keratinocyte migration.	Electroactive materials may influence wound repair through purinergic signaling and ionic communication, emphasizing the need to assess extracellular signaling downstream of stimulation.	H/C
Physiological pulsed direct-current electric fields [53]	Pulsed electrical stimulation → directional polarization/electrotaxis	Pulsed DC fields produced keratinocyte electrotaxis comparable to continuous DC stimulation while offering the potential to reduce electrochemical and electrothermal effects associated with continuous stimulation.	Waveform, pulse duration and duty cycle should be regarded as hydrogel/device design variables, not only field strength or conductivity.	C

Evidence level: H, human tissue/clinical physiology; LA, large-animal model; A, animal model; C, cellular/in vitro; G, genetic or molecular causal perturbation; BM, biomaterial-mediated intervention; NM, non-mammalian mechanistic model.

**Table 4:** Representative experimental evidence of mechanoredox, Mechanobioelectric, and redox–bioelectric coupling relevant to wound regeneration and hydrogel design.

Coupling Domain	Material/Stimulus	Coupling Mechanism	Experimental Evidence	Implications for Coupled Hydrogel Design	Coupling Evidence
Mechanoredox [33]	Mechanical injury; endogenous PIEZO1/DUOX2 signaling	PIEZO1 → DUOX2 → localized H_2_O_2_ → FER/cortactin → actin remodeling	Spatially confined H_2_O_2_ flashes were associated with lamellipodia formation, cellular retraction and migration, directly connecting wound-associated mechanosensing with redox-dependent cytoskeletal dynamics.	Mechanical properties of hydrogels may indirectly modify local redox signaling through mechanosensitive Ca^2+^/oxidase pathways; ROS scavenging should therefore preserve physiological mechanoredox signals.	Direct mechanistic
Mechanobioelectric [56]	Injectable gelatin-based conductive hydrogel containing SrTiO_3_ piezoelectric nanoparticles; body motion/bending	Mechanical deformation → piezoelectric polarization → local electrical stimulation → migration/proliferation	Mechanical bending generated output up to approximately 1 V and 0.5 nA; the material promoted fibroblast migration/proliferation and accelerated wound repair in mice.	Demonstrates how physiological motion can be converted directly into therapeutic electrical signals within the hydrogel, avoiding external power sources.	Direct material coupling
Mechanobioelectric [55]	Mechanically responsive conductive hydrogel	Mechanical deformation + electroactive/conductive network → endogenous-like electrical stimulation	The hydrogel was explicitly engineered around mechano-electric synergy and promoted migration, angiogenesis and wound closure rather than relying solely on passive conductivity.	Supports designing conductivity as a deformation-dependent functional output, rather than as a static material parameter.	Functional coupling
Mechanoredox–bioelectric [58]	BTO@Au-loaded thermoresponsive double-network hydrogel; ultrasound + NIR	Ultrasound → piezoelectric charge separation → ROS generation + local micro-electric field; thermoresponsive network → wound contraction	High-power ultrasound produced bactericidal ROS, whereas lower ultrasound power generated lower ROS levels together with a sustained local electric field that promoted fibroblast migration and angiogenesis. The hydrogel also actively contracted wound edges and activated FAK/AKT-associated repair.	One of the clearest examples of stage-dependent coupling, where the same piezoelectric component is tuned from ROS-mediated antimicrobial action toward electrical regenerative stimulation.	Strong integrated coupling
Bioelectric–redox [59]	Ag_2_Se@GelMA self-powered thermoelectric hydrogel	Temperature gradient → electrical field → voltage-gated Ca^2+^ channels → Ca^2+^/CaMKKβ/AMPK/NRF2	Thermoelectrically generated stimulation amplified the endogenous wound electric field, increased intracellular Ca^2+^ and activated the CaMKKβ/AMPK/NRF2 pathway, improving mitochondrial function and angiogenesis.	Demonstrates that an electrical output can regulate a classical redox-homeostasis pathway, providing direct rationale for designing electroactive hydrogels that modulate NRF2 signaling.	Direct signaling coupling
Redox–bioelectric [60]	Polyphenol/PDA–Fe–PEDOT conductive nanozyme hydrogel combined with vagus nerve electrical stimulation	Conductive/redox-active nanozyme + electrical stimulation → sustained ROS regulation + local/systemic inflammatory modulation	The hydrogel retained antioxidant activity during electrical stimulation, decreased intracellular ROS and protected cells from oxidative stress; coupling with vagus nerve stimulation enhanced collagen deposition, angiogenesis and nerve repair in diabetic wounds.	Particularly relevant to conductive polyphenol systems because redox chemistry and electrical transmission are incorporated within the same material architecture.	Functional; causality incomplete
Mechanoredox/piezoelectric [61]	Ultrasound-responsive piezoelectric hydrogel	Ultrasound mechanical energy → piezoelectric energy conversion → controlled ROS/CO generation + wound self-closure	Ultrasound energy was reconverted through the piezoelectric hydrogel to produce ROS/CO while simultaneously contributing to wound closure and infected chronic-wound repair.	Shows that mechanical energy input can directly regulate therapeutic redox output, an important strategy for on-demand redox-active hydrogels.	Direct energy coupling
Mechanobioelectric [55]	Axolotl-inspired biomechanical material + directional physiological electric field	Biomechanics + electric field → PIEZO1 modulation → keratinocyte migration	Reconstruction of both mechanical and electrical wound microenvironments accelerated re-epithelialization and promoted ordered ECM regeneration. Both cues converged on PIEZO1 and relieved its inhibitory effect on keratinocyte migration.	Provides unusually strong evidence that mechanical and electrical cues can converge on the same mechanosensitive molecular target rather than simply act in parallel.	Direct mechanistic coupling
Mechanobioelectric [62]	Motion-responsive piezoelectric hydrogel nanofibers	Physiological motion → piezoelectric electrical signal → intermediate-filament remodeling and metabolic regulation	A collagen-mimetic self-powered nanofibrous hydrogel converted motion into electrical stimulation and linked piezoelectric signaling with cytoskeletal/intermediate-filament remodeling and cellular energy metabolism during wound healing.	Extends mechanoelectric hydrogel design beyond migration alone to cytoskeletal organization and metabolic reprogramming.	Direct material coupling
Mechanoredox–bioelectric [63]	Hydrogel containing nitric oxide (NO)-donor-functionalized barium titanate (BT-dNO) nanoparticles (BT-dNO@Gel), activated by low-intensity focused ultrasound	LIFU mechanical energy → BT deformation → piezoelectric current + ROS; stimulus-triggered NO release → neuroimmune regulation	The same LIFU trigger produced localized piezoelectric current, controlled ROS generation and NO release. The system promoted M2-like macrophage responses, CGRP-mediated neuroimmune signaling and achieved 93% closure in diabetic wounds by day 14; regulated ROS also contributed to antibacterial activity in infected wounds.	Currently among the strongest examples of multidomain energy conversion within a hydrogel, because one external mechanical input simultaneously regulates electrical and redox outputs with defined biological consequences.	Strong triple coupling

**Table 5:** Representative recent hydrogels simultaneously exhibiting mechanical, redox, and electrical functions relevant to cutaneous wound regeneration.

Hydrogel/Network	Mechanical Domain	Redox Domain	Electrical Domain	Evidence of Integration	Classification
CMCS/THB/Cu/GO-BNN6 dynamic multifunctional hydrogel [67]	Stable mechanics, injectability and self-healing through dynamic network interactions	Polyphenol-associated antioxidant activity; NIR-controlled NO release	GO-containing electroconductive network	Mechanical, antioxidant and conductive functions demonstrated in one network, but causal dependence among the three domains was not established	Triple coexistence
Hep–PDA–rGO/PAM nanocomposite hydrogel [66]	rGO dispersion increased tensile/compressive strength; tensile strength ~250 kPa	PDA/rGO-containing system showed antioxidative wound-protective activity	Electronic conductivity up to 3.63 S m^−1^	The same Hep–PDA–rGO phase controls mechanical reinforcement and conductivity and contributes to redox function, but direct redox–electrical causality was not tested	Structurally integrated coexistence
PVA/alginate hydrogel containing sodium niobate [74]	Flexible hydrogel designed for wounds under repetitive movement	Photodynamic generation of bactericidal ROS	Movement-induced piezoelectric electric field	Mechanical movement acts as the energy source for electrical stimulation, while redox activity is independently photoactivated	Mechanoelectrical coupling + redox coexistence
BTO@Au incorporated thermoresponsive dual-network hydrogel [58]	Tough double network; temperature-sensitive softening and active contraction	Ultrasound-dependent ROS generation; high/low ROS regimes	Piezoelectric local micro-electric field; Au promotes charge transport/separation	A common mechanical input controls charge separation, ROS production and electrical output; network contraction independently modulates wound mechanics	Strong mechanoredox–electrical coupling
FGel-DA/SFMA hydrogel containing PDA–Fe–PEDOT conductive nanozymes [60]	Crosslinked soft hydrogel matrix provides conformal wound support	POD-like H_2_O_2_ conversion plus sustained antioxidant activity during ES	PEDOT-based conductive nanozyme transmits endogenous/exogenous electrical signals	Redox catalytic and electrical functions originate from the same PDA–Fe–PEDOT phase; electrical stimulation was tested together with redox performance	Redox–electrical coupling within a mechanically functional hydrogel
Chitosan-based conductive hydrogel coupled to P(VDF-TrFE)-BTO piezoelectric layer and CeO_2_@PDA components [78]	Conformal hydrogel architecture designed to maintain wound contact and withstand stimulation	CeO_2_/PDA-mediated redox regulation and antioxidant functionality	Conductive hydrogel receives ultrasound-generated piezoelectric field	Mechanical/ultrasound input produces electrical stimulation while the hydrogel simultaneously regulates oxidative stress	Multicomponent triple integration
Gelatin/PVA/grape-seed-extract/quaternary-chitosan hydrogel [75]	Mechanoadaptive network designed for deformable epidermal contact	Polyphenol-rich grape-seed extract (GSE) supplies antioxidant/anti-inflammatory function	Conductive network enables surface electromyography (sEMG) monitoring and bioelectronic interface	All three domains are incorporated and evaluated within a mechanoadaptive dressing, but direct redox-dependent electrical changes remain to be demonstrated	Triple coexistence with structural integration
Sodium alginate/tannic acid/PEDOT:PSS hydrogel with in situ Ca^2+^-mediated gelation [68]	Dynamic viscoelastic and mechanically stable alginate network	Tannic acid-associated antioxidant chemistry	PEDOT:PSS mixed electronic/ionic conduction; skin-relevant electrical behavior	Polyphenol chemistry, Ca^2+^ gelation and PEDOT:PSS transport coexist within the same network; promising platform for direct redox–electrical testing	Structurally integrated triple functionality
polyethyleneimine (PEI)/PVA hydrogel containing piezoelectric BT-dNO nanoparticles [63]	Adhesive, self-healing and degradable hydrogel; LIFU provides mechanical excitation	LIFU triggers ROS generation and NO release	Piezoelectric direct-current output and localized electric field	One mechanical input simultaneously controls electric current, ROS production and NO release; electron spin resonance (ESR) and electrical measurements directly characterize distinct outputs	Strong triple coupling

**Table 6:** Proposed hierarchy of evidence for cross-domain coupling in regenerative hydrogels.

Level	Definition	Minimum Experimental Requirement	Example of Acceptable Evidence	Insufficient Evidence
I. Coexistence	Multiple properties present in one formulation	Independent characterization of each property	(G′), antioxidant capacity, and conductivity measured separately	Describing independent properties as “coupled” or “synergistic”
II. Structural integration	A shared molecular/nanoscale phase contributes to ≥2 properties	Composition–structure–property relationships demonstrating a common structural origin	rGO simultaneously contributes to reinforcement and charge transport	Separate additives independently supplying each function
III. Directional coupling	Input in domain (X) generates measurable output in domain (Y)	(Y = f(X)) under controlled composition and environmental conditions	strain → voltage; ultrasound power → ROS generation	Comparing different formulations without varying the input
IV. Multidomain transduction	One input controls outputs in ≥2 different domains	Simultaneous or correlated measurement of multiple outputs	ultrasound → electrical potential + ROS	Independent electrical and antioxidant measurements

**Table 7:** Representative recent experimental systems illustrating different levels of cross-domain coupling.

System/Model	Primary Input	Cross-Domain Output	Evidence Level	Main Limitation for Proving Complete Coupling
Hep–PDA–rGO/PAM wound hydrogel [66]	Mechanical deformation	Resistance changes in conductive rGO network; redox functionality also present	Structural integration + mechanoelectrical sensitivity	Redox state was not systematically varied against electrical/mechanical properties
PEDOT–PDA silk microfiber/alginate–gelatin hydrogel; periodontal model [80]	Electron transfer/conductive phase incorporation	Catechol/quinone balance + conductivity + mechanical reinforcement	Direct redox–electrical interfacial evidence	Non-cutaneous model; reciprocity not demonstrated
Conductive gelatin/SrTiO_3_ wound hydrogel [56]	Mechanical bending	~1 V and ~0.5 nA piezoelectric output	Direct mechanoelectrical transduction	Long-term coupling during degradation requires further analysis
Mechano-electric conductive wound hydrogel [55]	Mechanical deformation	Electrically active regenerative response	Functional mechanoelectrical coupling	Molecular relation between deformation and electrical transport incompletely resolved
BTO@Au thermoresponsive double-network hydrogel [58]	Ultrasound/NIR	Piezoelectric field + power-dependent ROS + thermal contraction	Strong multidomain feed-forward coupling	Reciprocal effect of redox state on mechanics/electrical output not established
PDA–Fe–PEDOT conductive nanozyme wound hydrogel [60]	Electrical stimulation/redox environment	Conductive transmission + catalytic/antioxidant activity	Redox–electrical structural and functional integration	Quantitative conductivity–redox-state dependence lacking
Ag_2_Se@GelMA thermoelectric wound hydrogel [59]	Temperature gradient	Electrical output → Ca^2+^/CaMKKβ/AMPK/NRF2	Electrical-to-biological-redox coupling	Redox coupling occurs mainly in cells rather than within material network
Alginate/rGO/proanthocyanidin redox-capacitive hydrogel [81]	Interfacial electrochemical/redox processes	Impedance- and charge-storage-related response	Redox–electrical structural integration	Reciprocal perturbation experiments remain desirable
Biomechanical + directional electric-field wound material [55]	Mechanical and electrical cues	PIEZO1-mediated migration	Coupling at material–cell interface	Redox component not evaluated
BT-dNO@Gel + LIFU [63]	Ultrasound mechanical input	Piezoelectric microcurrent + ROS + NO	Strong multidomain feed-forward coupling	Closed-loop material feedback remains unproven

**Table 8:** Recommended experimental criteria for demonstrating specific coupling modes in regenerative hydrogels.

Coupling Mode	Input That Should Be Controlled	Required Material-Level Output	Preferred Complementary Characterization	Key Controls	Strong Evidence
Mechanical → electrical	Stress, strain, loading frequency; hydration/swelling state	Voltage, current, charge, resistance/impedance; σ_ion_, σ_e_, and σ_total_ when mixed conduction is present	Piezoresponse force microscopy; dielectric characterization; in situ strain-dependent EIS with equivalent-circuit analysis; DC polarization or adapted Hebb–Wagner analysis; four-terminal resistance when feasible	Non-piezoelectric/non-conductive controls; ionic-only control; constant electrolyte composition and temperature; instantaneous geometry correction under strain/swelling; electrode-contact control	Reproducible electrical output scaling with mechanical input, with experimental resolution of the ionic and/or electronic origin of the response
Electrical → mechanical	Applied potential/current	(G′), modulus, strain, contraction, relaxation	Rheology under electrical stimulation	Joule-heating control	Reversible electrical control of mechanics
Mechanical → redox	Stress, strain, ultrasound power	ROS rate/speciation, redox potential	ESR, defined ROS probes, electrochemistry	Thermal/cavitation controls	ROS output scaling with mechanical input
Redox → mechanical	H_2_O_2_, GSH, potential, oxidation state	(G′), (G″), relaxation, adhesion, swelling	Spectroscopy of bond state	pH/osmolarity controls	Mechanics changes with verified redox transformation
Redox → electrical	Oxidation potential/state	Conductivity, (R_ct_), capacitance, impedance; σ_ion_, σ_e_, σ_total_, and corresponding transport fractions when mixed conduction occurs	XPS, Raman, CV, spectroelectrochemistry; EIS equivalent-circuit analysis; DC polarization/Hebb–Wagner-type validation	Hydration, swelling, ionic-strength, pH and geometry controls; electronically non-conductive and/or ionic-only controls where feasible	Electrical transport tracks independently verified redox state, and changes can be assigned to ionic and/or electronic transport rather than hydration or electrolyte effects
Electrical → redox	Potential/current	ROS flux, catechol/quinone ratio, catalytic activity	ESR, XPS, electrochemistry	Thermal controls	Electrical input quantitatively changes redox chemistry
Triple coupling	One primary stimulus	≥2 simultaneous cross-domain outputs	Synchronized rheology/electrochemistry/ROS analysis	Matched single-domain controls	Defined stimulus controls mechanical, electrical and redox states
Reciprocal coupling	Cyclic or sequential perturbation	Feedback-modified second response	Time-resolved multimodal measurements	History-matched controls	Output from first cycle modifies transduction in subsequent cycles

## Data Availability

No new data were created or analyzed in this study. Data sharing is not applicable to this article.

## Abbreviations

The following abbreviations are used in this manuscript:

8-OHdG	8-Hydroxy-2′-deoxyguanosine
AAV	Adeno-associated virus
ABTS	2,2′-Azino-bis(3-ethylbenzothiazoline-6-sulfonic acid)
AC	Alternating current
AKT	Protein kinase B
AMPK	AMP-activated protein kinase
Arg1	Arginase 1
ATP	Adenosine triphosphate
BNN6	N,N′-Di-sec-butyl-N,N′-dinitroso-1,4-phenylenediamine
BTO	Barium titanate (BaTiO_3_)
BT-dNO	Nitric oxide donor-loaded barium titanate nanoparticles
CaMKKβ	Calcium/calmodulin-dependent protein kinase kinase beta
CCL2	C-C motif chemokine ligand 2
CCL24	C-C motif chemokine ligand 24
CGRP	Calcitonin gene-related peptide
CMCS	Carboxymethyl chitosan
CO	Carbon monoxide
CV	Cyclic voltammetry
DC	Direct current
DPPH	2,2-Diphenyl-1-picrylhydrazyl
DUOX	Dual oxidase
DUOX2	Dual oxidase 2
ECM	Extracellular matrix
EGF	Epidermal growth factor
EGR1	Early growth response 1
EIS	Electrochemical impedance spectroscopy
EN1	Engrailed-1
ERK	Extracellular signal-regulated kinase
ESR	Electron spin resonance
FAK	Focal adhesion kinase
FER	FER tyrosine kinase
FGel-DA	Dopamine-grafted fish gelatin
FOXO1	Forkhead box O1
FRAP	Ferric reducing antioxidant power
GB	Graphene oxide–BNN6 nanocomposite
GelMA	Gelatin methacryloyl
GO	Graphene oxide
GSE	Grape-seed extract
GSH	Reduced glutathione
GSSG	Oxidized glutathione
HA	Hyaluronic acid
HDM2	Human double minute 2
Hep	Heparin
IKKα	IκB kinase alpha
KEAP1	Kelch-like ECH-associated protein 1
LIFU	Low-intensity focused ultrasound
MAPK	Mitogen-activated protein kinase
MCP-1	Monocyte chemoattractant protein-1
MFGE8	Milk fat globule-EGF 8
MMP-8	Matrix metalloproteinase-8
MMP12	Matrix metalloproteinase-12
MnSOD	Manganese superoxide dismutase
NADPH	Nicotinamide adenine dinucleotide phosphate
NIR	Near-infrared
NO	Nitric oxide
NOX4	NADPH oxidase 4
NQO1	NAD(P)H quinone dehydrogenase 1
NRF2	Nuclear factor erythroid 2-related factor 2
OAT	Ornithine aminotransferase
P2Y	P2Y purinergic receptor
PAM	Polyacrylamide
PANI	Polyaniline
PDA	Polydopamine
PEDOT	Poly(3,4-ethylenedioxythiophene)
PEI	Polyethyleneimine
PFM	Piezoresponse force microscopy
PI3K	Phosphoinositide 3-kinase
PIEZO1	Piezo-type mechanosensitive ion channel component 1
P(PVDF-TrFE)	Poly(vinylidene fluoride-co-trifluoroethylene)
PPy	Polypyrrole
PSS	Poly(styrene sulfonate)
PTEN	Phosphatase and tensin homolog
PVA	Poly(vinyl alcohol)
PYCR1	Pyrroline-5-carboxylate reductase 1
Rac1	Rac family small GTPase 1
rGO	Reduced graphene oxide
RNS	Reactive nitrogen species
ROCK	Rho-associated coiled-coil-containing protein kinase
ROS	Reactive oxygen species
SFMA	Methacrylated silk fibroin
siRNA	Small interfering RNA
SIVA1	SIVA1 apoptosis-inducing factor
sEMG	Surface electromyography
STAT3	Signal transducer and activator of transcription 3
TAZ	Transcriptional coactivator with PDZ-binding motif
TEAD	TEA domain transcription factor
TGF-β	Transforming growth factor beta
THB	2,3,4-Trihydroxybenzaldehyde
VEGF	Vascular endothelial growth factor
VEGFR	Vascular endothelial growth factor receptor
XPS	X-ray photoelectron spectroscopy
YAP	Yes-associated protein
